# Guideline of the Brazilian Society of Cardiology on Telemedicine in Cardiology - 2019

**DOI:** 10.5935/abc.20190205

**Published:** 2019-11

**Authors:** Marcelo Antônio Cartaxo Queiroga Lopes, Gláucia Maria Moraes de Oliveira, Antonio Luiz Pinho Ribeiro, Fausto J. Pinto, Helena Cramer Veiga Rey, Leandro Ioschpe Zimerman, Carlos Eduardo Rochitte, Fernando Bacal, Carisi Anne Polanczyk, Cidio Halperin, Edson Correia Araújo, Evandro Tinoco Mesquita, José Airton Arruda, Luis Eduardo Paim Rohde, Max Grinberg, Miguel Moretti, Paulo Ricardo Avancini Caramori, Roberto Vieira Botelho, Andréa Araújo Brandão, Ludhmila Abrahão Hajjar, Alexandre Fonseca Santos, Alexandre Siciliano Colafranceschi, Ana Paula Beck da Silva Etges, Bárbara Campos Abreu Marino, Bruna Stella Zanotto, Bruno Ramos Nascimento, Cesar Rocha Medeiros, Daniel Vitor de Vasconcelos Santos, Daniela Matos Arrowsmith Cook, Eduardo Antoniolli, Erito Marques de Souza Filho, Fábio Fernandes, Fabio Gandour, Francisco Fernandez, Germano Emilio Conceição Souza, Guilherme de Souza Weigert, Iran Castro, Jamil Ribeiro Cade, José Albuquerque de Figueiredo Neto, Juliano de Lara Fernandes, Marcelo Souza Hadlich, Marco Antonio Praça Oliveira, Maria Beatriz Alkmim, Maria Cristina da Paixão, Maurício Lopes Prudente, Miguel A. S. Aguiar Netto, Milena Soriano Marcolino, Monica Amorim de Oliveira, Osvaldo Simonelli, Pedro A. Lemos Neto, Priscila Raupp da Rosa, Renato Minelli Figueira, Roberto Caldeira Cury, Rodrigo Coelho Almeida, Sandra Regina Franco Lima, Silvio Henrique Barberato, Thiago Inocêncio Constancio, Wladimir Fernandes de Rezende

**Affiliations:** 1 Hospital Alberto Urquiza Wanderley, João Pessoa, PB - Brazil; 2 Universidade Federal do Rio de Janeiro (UFRJ), Rio de Janeiro, RJ - Brazil; 3 Universidade Federal de Minas Gerais (UFMG), Belo Horizonte, MG - Brazil; 4 Universidade de Lisboa, Lisboa - Portugal; 5 Instituto Nacional de Cardiologia do Rio de Janeiro, Rio de Janeiro, RJ - Brazil; 6 Hospital de Clínicas de Porto Alegre, Porto Alegre, RS - Brazil; 7 Instituto do Coração (InCor) do Hospital das Clínicas da Faculdade de Medicina da Universidade de São Paulo (USP), São Paulo, SP - Brazil; 8 Universidade Federal do Rio Grande do Sul (UFRGS), Porto Alegre, RS - Brazil; 9 Instituto de Avaliação de Tecnologias em Saúde (IATS), Porto Alegre, RS - Brazil; 10 Hospital Ernesto Dornelles, Porto Alegre, RS - Brazil; 11 Banco Mundial, Brasília, DF - Brazil; 12 Universidade Federal Fluminense (UFF), Rio de Janeiro, RJ - Brazil; 13 Universidade Federal do Espírito Santo (UFES), Vitória, ES - Brazil; 14 Hospital São Lucas da Pontifícia Universidade Católica do Rio Grande do Sul (PUCRS), Porto Alegre , RS - Brazil; 15 Instituto do Coração do Triângulo (ICT), Uberlândia, MG - Brazil; 16 International Telemedical Systems do Brasil (ITMS), Uberlândia, MG - Brazil; 17 Universidade do Estado do Rio de Janeiro (UERJ), Rio de Janeiro, RJ - Brazil; 18 Hospital Pró-Cardíaco, Rio de Janeiro, RJ - Brazil; 19 Hospital Madre Teresa, Belo Horizonte, MG - Brazil; 20 Pontifícia Universidade Católica de Minas Gerais (PUCMG), Belo Horizonte, MG - Brazil; 21 Hospital das Clínicas da Universidade Federal de Minas Gerais (UFMG), Belo Horizonte, MG - Brazil; 22 Hospital Copa D'Or, Rio de Janeiro, RJ - Brazil; 23 Hospital Copa Star, Rio de Janeiro, RJ - Brazil; 24 Hospital dos Servidores do Estado do Rio de Janeiro, Rio de Janeiro, RJ - Brazil; 25 Hospital Santa Ana, Porto Alegre, RS - Brazil; 26 Universidade Federal Rural do Rio de Janeiro, Seropédica, RJ - Brazil; 27 UMC Imagem, Uberlândia, MG - Brazil; 28 Universidade de Brasília (UnB), Brasília, DF - Brazil; 29 Universidade de São Paulo (USP), São Paulo, SP - Brazil; 30 Americas Medical City, Rio de Janeiro, RJ - Brazil; 31 Conexa Saúde, Rio de Janeiro, RJ - Brazil; 32 Instituto de Cardiologia do Rio Grande do Sul, Porto Alegre, RS - Brazil; 33 Fundação Universitária de Cardiologia, Porto Alegre, RS - Brazil; 34 Hospital Santa Marcelina, São Paulo, SP - Brazil; 35 Universidade Federal do Maranhão, São Luís, MA - Brazil; 36 Instituto de Ensino e Pesquisa José Michel Kalaf, Campina, SP - Brazil; 37 Fleury Medicina e Saúde, Rio de Janeiro, RJ - Brazil; 38 Rede D'Or, Rio de Janeiro, RJ - Brazil; 39 Unimed-Rio, Rio de Janeiro, RJ - Brazil; 40 Hospital ENCORE, Goiânia, GO - Brazil; 41 BrasilSaúde Cia de Seguros, Rio de Janeiro, RJ - Brazil; 42 Conselho Regional de Medicina do Estado de São Paulo, São Paulo, SP - Brazil; 43 Instituto Paulista de Direito Médico e da Saúde (IPDMS), Ribeirão Preto, SP - Brazil; 44 Hospital Israelita Albert Einstein, São Paulo, SP - Brazil; 45 Hospital Sírio Libanês, São Paulo, SP - Brazil; 46 DASA, São Paulo, SP - Brazil; 47 Medportal, Rio de Janeiro, RJ - Brazil; 48 Sfranco Consultoria Jurídica, São José dos Campos, SP - Brazil; 49 CardioEco-Centro de Diagnóstico Cardiovascular, Curitiba, PR - Brazil; 50 Quanta Diagnóstico e Terapia, Curitiba, PR - Brazil; 51 Fundação Oswaldo Cruz (Fiocruz), Rio de Janeiro, RJ - Brazil; 52 UMC Tecnologia, São Paulo, SP - Brazil

**Table:** Grades of recommendation and levels of evidence in this update were applied according to the following standards:

Classes (grades) of recommendation	
Grade I	Conditions for which there is conclusive evidence or, in the absence of conclusive evidence, a general consensus that the procedure is safe and useful/effective.
Grade IIa	Conditions for which there is conflicting evidence and/or divergent opinions regarding the procedure's safety and usefulness/effectiveness. Weight or evidence/opinion in favor of the procedure. Received approval by most studies/experts.
Grade IIb	Conditions for which there is conflicting evidence and/or divergent opinions regarding the procedure's safety and usefulness/effectiveness. Safety and usefulness/effectiveness are less well established, with no prevailing opinions in favor.
Grade III	Conditions for which there is evidence and/or consensus that the procedure is not useful/effective and in some cases may be potentially harmful.

**Table:** 

Levels of evidence	
Level A	Data obtained from multiple, concordant, large randomized trials, and/or robust meta-analysis of randomized clinical trials.
Level B	Data obtained from less robust meta-analysis, from a single randomized trial, or from nonrandomized (observational) trials.
Level C	Data obtained through a consensus of expert opinions.

**Table:** 

Declaration of potential conflict of interests of authors/collaborators of theGuideline of the Brazilian Society of Cardiology on Telemedicine in Cardiology - 2019 If, within the last 3 years, the author/collaborator of the guideline:							
Names of guideline collaborators	Participated in clinical and/or experimental studies sponsored by pharmaceutical or equipment companies related to this guideline	Spoke at events or activities sponsored by industry related to this guideline	Was (is) a member of a board of advisors or a board of directors of a pharmaceutical or equipment industry	Participated in normative committees of scientific research sponsored by industry	Received personal or institutional funding from industry	Wrote scientific papers in journals sponsored by industry	Owns stocks in industry
Alexandre Fonseca Santos	No	No	No	No	No	No	No
Alexandre Siciliano Colafranceschi	No	No	No	No	No	No	No
Ana Paula Beck da Silva Etges	No	No	No	No	No	No	No
Andréa Araújo Brandão	No	No	No	No	No	No	No
Antonio Luiz Pinho Ribeiro	No	No	No	No	No	No	No
Bárbara Campos Abreu Marino	No	No	No	No	No	No	No
Bruna Stella Zanotto	No	No	No	No	No	No	No
Bruno Ramos Nascimento	No	No	No	No	No	No	No
Carisi Anne Polanczyk	No	No	No	No	No	No	No
Carlos Eduardo Rochitte	No	No	No	No	No	No	No
Cesar Rocha Medeiros	No	No	No	No	No	No	No
Cidio Halperin	Apple	No	No	No	No	No	No
Daniel Vitor de Vasconcelos Santos	No	No	No	No	No	No	No
Daniela Matos Arrowsmith Cook	No	No	No	No	No	No	No
Edson Correia Araújo	No	No	No	No	No	No	No
Eduardo Antoniolli	No	No	No	No	No	No	No
Erito Marques de Souza Filho	No	No	No	No	No	No	No
Evandro Tinoco Mesquita	No	No	No	No	No	No	No
Fábio Fernandes	No	No	No	No	No	No	No
Fabio Gandour	No	No	No	No	No	No	No
Fausto J. Pinto	No	No	No	No	No	No	No
Fernando Bacal	No	No	No	No	No	No	No
Francisco Fernandez	No	No	No	No	No	No	No
Germano Emilio Conceição Souza	No	No	No	No	No	No	No
Gláucia Maria Moraes de Oliveira	No	No	No	No	No	No	No
Guilherme de Souza Weigert	No	No	Conexa Saúde	No	Conexa Saúde	No	Conexa Saúde
Helena Cramer Veiga Rey	No	No	No	No	No	No	No
Iran Castro	No	No	No	No	No	No	No
Jamil Ribeiro Cade	No	No	No	No	No	No	No
José Airton de Arruda	No	No	No	No	No	No	No
José Albuquerque de Figueiredo Neto	No	No	No	No	No	No	No
Juliano Lara Fernandes	No	No	No	No	No	No	Hypera Pharma, Grupo Biotoscana
Leandro Ioschpe Zimerman	No	No	No	Pfizer	Bayer, Pfizer, Biotronik	No	No
Ludhmila Abrahão Hajjar	No	No	No	No	No	No	No
Luis Eduardo Paim Rohde	No	No	No	No	No	No	No
Marcelo Antônio Cartaxo Queiroga Lopes	No	No	No	No	No	No	No
Marcelo Souza Hadlich	No	No	No	No	No	No	No
Marco Antonio Praça Oliveira	No	No	No	No	No	No	No
Maria Beatriz Alkmim	No	No	No	No	No	No	No
Maria Cristina da Paixão	No	No	No	No	No	No	No
Maurício Lopes Prudente	No	No	No	No	No	No	No
Max Grinberg	No	No	No	No	No	No	No
Miguel A. S. Aguiar Netto	No	No	No	No	No	No	No
Miguel Antonio Moretti	No	No	No	No	No	No	No
Milena Soriano Marcolino	No	No	No	No	No	No	No
Monica Amorim de Oliveira	No	No	No	No	No	No	No
Osvaldo Simonelli	No	No	No	No	No	No	No
Paulo Ricardo Avancini Caramori	No	No	Medtronic	SciTech, Biotronik	No	No	No
Pedro A. Lemos Neto	No	No	No	No	No	No	No
Priscila Raupp da Rosa	No	Aruba/Kapersky	No	No	No	No	No
Renato Minelli Figueira	No	No	No	No	No	No	No
Roberto Caldeira Cury	No	No	No	No	No	No	No
Roberto Vieira Botelho	No	No	No	No	No	No	No
Rodrigo Coelho de Almeida	No	No	No	No	No	No	No
Sandra Regina Franco Lima	No	No	No	No	No	No	No
Silvio Henrique Barberato	No	No	No	No	No	No	No
Thiago Inocêncio Constancio	No	No	No	No	No	No	No
Wladimir Fernandes de Rezende	No	No	No	No	No	No	No

## Presentation

In due time, the Brazilian Society of Cardiology decided to create a guideline on telemedicine applied to cardiology, also known as telecardiology. According to the Pan American Health Organization (PAHO) and the World Health Organization (WHO), telemedicine is “The delivery of health care services, where distance is a critical factor, by all health care professionals using information and communication technologies for the exchange of valid information for diagnosis, treatment, and prevention of disease and injuries, research and evaluation, and for the continuing education of health care providers, all in the interests of advancing the health of individuals and their communities.” Such a seemingly simple and altruistic definition carries a wide range of potential implications at various levels, from an ethical point of view to a potential impact on clinical practice and outcomes. Hence, the importance of guidelines, organized by the medical community through scientific societies, in offering to all of those involved in the process a reference based, as much as possible, on expert opinion, current scientific evidence, and on respect for medical ethical and deontological values.

Considering that cardiovascular diseases are the main cause of morbidity and mortality in the 21st century in Brazil and worldwide, the opportunity to use instruments to allow more effective actions in the prevention, diagnosis, treatment, and follow-up of these diseases paves the way to very relevant perspectives of better care for the populations and communities that we serve. At the same time, bioethical aspects and consequences should never be neglected, as they can (and should) undermine programs that, disguised as “medical,” fail to meet these ethical requirements. Therefore, regulated operating models based on guidelines organized by medical-scientific authorities are fundamental in striking a balance.

The introduction and implementation of new digital technologies are favoring the emergence of new methodologies (many still experimental) aimed at improving the capacity of intervention on individual patients and allowing for more customized care. We are experiencing what Eric Topol^1^ in his latest book, “Deep Medicine: How Artificial Intelligence Can Make Healthcare Human Again,” called the “Fourth Industrial Age” comprising artificial intelligence, robotics, and big data that will have a great impact on the way we live and see ourselves as human beings. If this is very positive at first sight, it is also true that it is not devoid of risk, particularly in the way that we approach or will approach the patient. Therefore, one must not forget the Hippocratic principle: “It is far more important to know what person the disease has than what disease the person has.” In fact, when we are sick, we all want to have our doctor - and not a computer - taking care of us and offering us a word of comfort and confidence.

Therefore, we must think smartly about how to apply to human benefit this impressive array of elements that have opened up frontiers that were unfathomable just a few years ago. Telemedicine - or telecardiology - can indeed play a very important role, particularly when this may be the only available resource. However, its use must be properly delineated to prevent abuse and misuse. The present document and guideline was prepared for this purpose. This complete document offers a detailed review of the regulation of telemedicine in Brazil, defines the meaning of a geographically remote area, and describes the fundamentals of telemedicine and the secure grounds for its transmission.

This document also offers up-to-date information on current evidence and applications of so-called teleconsultation, telediagnosis, and telemonitoring, and reflects on how telemedicine can provide technology-based medical services, with artificial intelligence playing a key role. The document also includes the economic assessment and budgetary impact of incorporating telemedicine in cardiology in Brazil and telemedicine in supplementary health, and - in one of the most important chapters - presents the ethical and legal aspects of telemedicine. Finally, the document includes a set of recommendations intended to be practical and adapted to the Brazilian perspective.

The result is a guideline perfectly aligned with the WHO guidelines on the principle that the implementation of telemedicine must be properly planned and should predict situations like the feasibility of network coverage for technology access in remote locations, construction of a legal and judicial structure for the implementation, budgetary impact and cost-effectiveness assessment of the implementation of each stage of the project, and development of indicators of the clinical continuum of applicability for user safety. As the president-elect of the World Heart Federation, I see this as a model document in terms of how it was planned and implemented, as well as in its content, reflecting the current evidence and perspective of the main scientific players in the area. As such, I think it will become a historical document, a milestone in the responsible introduction of telemedicine-telecardiology in clinical practice, in this case, applied to Brazil, but which can serve as an example for others globally, contributing to decrease the burden of cardiovascular diseases worldwide.

Lisbon, June 2019.

**Prof. Fausto J. Pinto, FESC, FACC**

President-elect, World Heart Federation (WHF)

Past President, European Society of Cardiology (ESC)

University of Lisbon, Portugal

## Introduction

For more than 26 years now, starting after the publication of the Consensus on Severe Heart Disease in 1993,^2^the Brazilian Society of Cardiology (SBC) has been regularly issuing guidelines on most diverse topics, guiding the practice of cardiology in Brazil. In 1999, the Brazilian Federal Council of Medicine (CFM)^3^partnered with the Brazilian Medical Association (AMB) and, aiming to support medical decision making and optimize patient care, started a process along with specialty societies for the development of Medical Guidelines based on current scientific evidence. Thus, the commitment of SBC precedes the initiative by AMB and fulfills one of the society’s objectives, described in the society’s bylaws.

Resolution 1.642/2002,^4^passed by the CFM to preserve the autonomy of the physician, defined that, in their relationship with physicians and beneficiaries, health insurance and group medical companies, medical cooperatives, self-management companies, and other companies offering direct care or care mediated by medical-hospital services should only adopt medical guidelines or protocols prepared by Brazilian specialty societies along with the AMB. Within this context,^5^the CFM initiated discussions in 2018 to update the regulations of telemedicine.

Telemedicine can be defined as the application of information and communication technologies to health care with the goal of offering, in a broad concept, health-related services ranging from primary care to robotic surgery and education, expanding coverage to remote areas in a country with continental dimensions.

The Pan American Health Organization (PAHO) and the WHO define telemedicine as “The delivery of health care services, where distance is a critical factor, by all health care professionals using information and communication technologies for the exchange of valid information for diagnosis, treatment, and prevention of disease and injuries, research and evaluation, and for the continuing education of health care providers, all in the interests of advancing the health of individuals and their communities.” The PAHO estimates that one third of the population in the Americas has no access to health care and that 800,000 additional health care professionals would be needed to meet the needs in the region.^6^If applied in its broad context, telemedicine could allow access and reduce inequality for this population by providing supposedly cost-effective quality services, especially considering the increased prevalence and mortality from chronic noncommunicable diseases (NCDs) in low- and middle-income countries like Brazil. Added to this context is the aging and increasing disease rate of the Brazilian population, which makes telemedicine an ideal tool to face the contemporary challenges of universal health care systems.^7^

Beyond the vast possibilities and applications of telemedicine, rigorous evaluations of telemedicine projects must be undertaken, not only because all health care systems face financial sustainability challenges beyond investments in health care interventions, but also because of the limited clinical evidence available, especially in the current order of value-based medicine. This topic of utmost importance has been the subject of several publications by the WHO. Examples of that include the Digital Health Atlas,^8^ a global virtual platform to support governments in monitoring and coordinating digital health activities; “BeHe@lthy, BeMobile” (BHBM),^9^for the prevention and control of NCDs; and mHealth Assessment and Planning for Scale (MAPS), a manual for digital health monitoring and evaluation^10^to enhance digital health research and implementation; among others. These documents culminated in the publication by the WHO of the first guideline on digital health interventions on April 17, 2019.^11^

In addition to updating the guideline on telemedicine applicable to cardiology published in 2015, the main objective of the present guideline is to answer the following questions: Is there legal and ethical support for the application of telemedicine in Brazil? Are there technical conditions for the application of telemedicine in the country? What is the priority of incorporating telemedicine into the health care system? For which modalities is there good quality scientific evidence to support this practice? For modalities supported by solid evidence, does cost effectiveness justify this application? What would be the budgetary impact? Is the Brazilian health care system prepared to provide comprehensive care?

This guideline, which is in line with the WHO guidelines,^11^ advocates that the implementation of telemedicine should be a planned process that provides feasibility of the network coverage in remote locations, elaboration of the legal and judicial bases for its implementation, budgetary impact and cost-effectiveness assessment of each stage of the project, and development of clinical continuum indicators of the applicability for the safety of the beneficiaries. Telemedicine can be a potential tool in improving health care services but is not exempt from risks and challenges related to its implementation and from the evaluation of the real impact of its benefits.

In the final chapter, the authors present a summary of recommendations based on current evidence, in an attempt to guide the discussions that will certainly permeate the democratization of comprehensive health care services, especially the actions involving telemedicine as a tool to expand the universality and integrality of the Brazilian Unified Health System (SUS), recommendations that also extend to supplementary health care.

Brazil, June 2019.

**Dr. Marcelo Antônio Cartaxo Queiroga, FESC, TEC-SBC**

President-elect of the Brazilian Society of Cardiology (Sociedade Brasileira de Cardiologia - SBC)

Director of the Department of Interventional Cardiology, Alberto Urquiza Wanderley Hospital, João Pessoa, PB, Brazil Member of the Paraíba State Academy of Medicine

**Dr. Gláucia Maria Moraes de Oliveira, FACC, FESC, TEC-SBC**

Associate Professor of Cardiology at the Federal University of Rio de Janeiro (Universidade Federal do Rio de Janeiro - UFRJ)

Coordinator of the Postgraduate Cardiology Program at UFRJ, Rio de Janeiro, RJ, Brazil

President of the Federation of the Cardiology Societies of the Portuguese-Speaking Countries (2015-2016)

## 1. Fundamentals of Telemedicine: Concepts, Bioethical Aspects, Legislation and Regulation, Applicability in Brazil, and Artificial Intelligence

### 1.1. Fundamentals of Telemedicine

In May 2005, Ministers of Health from 192 countries members of the World Health Organization (WHO) approved the Resolution on eHealth,^12^ which recognized for the first time the importance of information and communication technologies (ICTs) applied to health - digital health or eHealth - “reinforcing the fundamental human rights by increasing and improving equity, solidarity, quality of life, and quality of care.”

The Brazilian Ministry of Health defines the following areas of telehealth application:^13^

**Innovation in digital health and telehealth**

Innovation in digital health is transversal to telehealth initiatives and seeks to explore via ICT new ideas to solve chronic problems with difficult solutions by usual methods. It must start with the population’s health care needs.

**Teleconsulting**

Registered consultation between health care workers, professionals, and managers using two-way telecommunication instruments in order to answer questions about clinical procedures, health care actions, and suggestions related to the work process in health care. Teleconsulting can occur in real time or by offline messaging.

**Telediagnosis**

Autonomous service using ICT to deliver diagnostic support services (*e.g.,* remote evaluation of diagnostic tests) to facilitate access to specialized services. The use of telediagnosis seeks to reduce the time to diagnosis by enabling treatment for predictable complications through early diagnosis.

**Telemonitoring**

Remote monitoring of patients’ health and/or disease parameters through ICT. Monitoring may include clinical data collection, transmission, processing, and management by a health care professional using an electronic system.

**Teleregulation**

Set of actions in regulatory systems for evaluation of adequate responses to existing demands, promoting equity and access to services, and enabling health care access. Teleregulation also includes the evaluation and planning of actions to provide regulatory operational intelligence to management teams. The objective of teleregulation is to potentiate primary health care services, thus enabling the qualification and reduction of wait for specialized care.

**Tele-education**

Availability of interactive educational materials on health-related topics delivered remotely through ICT and focused on professional education across activity areas.

### 1.2. Types of Intervention in Telehealth

Synchronous video conference: modality of remote interaction via live conference between primary care and medical specialty services.

Asynchronous video conference (“store and forward”): use of a storage system to forward diagnostic images, vital signs, and/or video clips along with patients’ data for later review by a specialist. Provides diagnostic and treatment support for the primary care system.

Remote monitoring: use of equipment to remotely collect and forward patients’ data to a hospital or monitoring center for interpretation. These (wearable) devices monitor remotely a variety of indicators ranging from specific vital signs (heart rate, blood pressure [BP], and blood glucose) to other indicators.

Mobile health (mHealth): defined as a medical and public health care practice supported by mobile devices like cell phones, monitoring devices, personal digital assistants (PDAs), and other wireless devices.^14^

The goals of telemedicine include:

Remote assistance: teleconsultation, telediagnosis or diagnostic telemonitoring, remote patient monitoring and/or treatment;

Administrative management of patient care: request of diagnostic tests, medical prescriptions, and actions related to service reimbursement;

Remote qualification of human resources to facilitate continuing education programs;

Network collaborative clinical research: use of ICT to share and disseminate best practices and generate knowledge.

### 1.3. Safe Bases for Data Transmission

Information safety is fundamental for data transmission, and two immediate effects must be considered: a) understanding of the critical value of data storage and use, and b) possible implications for individuals and organizations of violating safety and compliance standards.

The European General Data Protection Regulation (GDPR) and the Brazilian General Data Protection Act (*Lei Geral de Proteção de Dados*, LGPD) impose heavy fines and sanctions for improper access to information under their custody.

The following sections list the main requirements for establishing appropriate safety policies.^15^

### 1.4. Data Protection and Confidentiality

For proper information protection, the safety of the systems must be ensured, reducing vulnerabilities and preventing improper access and breach of confidentiality. Authorizations and hierarchical levels for access to information must be clearly determined.^16^

The policy related to information access and confidentiality must be reported in a document signed by the users defining the a) scope of data that can be accessed and b) legal implications and sanctions eventually applied to users in case of violation of the agreed rules.

Misuse of technological installations is directly related to the safety of the environments under the responsibility of ICT teams. Strict policies must be adopted in terms of access to physical facilities, data networks, operating systems, and databases and their applications. A valuable framework to provide an understanding of the control of these environments can be found in the document “Access Control Example Policy” (Health and Social Care Information Centre, 2017).^16^

The recommended standard for data transmission in Brazil follows the set of rules determined by the Health Insurance Portability and Accountability Act (HIPAA).17 This set of norms has proven robust enough to ensure the safety of the transferred data and is recommended as the benchmark for data transfer practices. The CFM Resolution 2.227/2018, now revoked, set the standard that would meet the desirable requirements: “Use of a proprietary or an open-source electronic/digital information registration system that captures, stores, presents, transfers, or prints digitally identifiable health information and is fully compliant with the requirements of Safety Assurance Level 2 (*Nível de Garantia de Segurança 2*, NGS2) and the ICP-Brazil standard.”

According to these standards, stored data (“at rest”; “in transit”) must be encrypted for transfer. One of the essential practices for data security is to maintain the tools required to encrypt and decrypt information in environments other than the original storage locations.^18-20^

In addition to ensuring information security, HIPAA rules offer extensive documentation for data encryption and transfer, facilitating the work of development teams. Of note, national public data cannot be stored in cloud systems hosted outside the country.^21-22^

### 1.5. Bioethical Aspects

Initiatives to provide remote health care through telemedicine date back to the 19th century. Cardiology was a pioneer in this initiative, with the description by Einthoven in 1906 of a transtelephonic electrocardiographic transmission from the academic hospital to the physiology laboratory at Leiden University, a few miles away.^23^ The big boost in the development of telemetry was by the North American Space Agency (NASA) in astronaut monitoring.^24,25^

However, the incorporation of telemedicine, as currently conceived, is contemporaneous^24-29^ and linked to the traditional notion that the preservation of the social value of medicine depends on content flow. Any modality of telecommunication holds both constructive and destructive potentials that trigger contradictions in terms of values and rules of moral code related to bedside medical practice. Ambivalence is welcome in medicine, which according to Osler (1849-1919), is the science of uncertainty and the art of probability.^28^ Telemedicine is not immune to the pendular movements of the variety of methods addressing health needs.

Bedside practice faces dilemmas inherent to the diversity of the human condition.^30^ Physicians and patients face external and/or internal challenges without a single and simple solution. Any option to be considered must be judiciously expressed, clarified, and adjusted to be validated for the conceptual and individual context of the clinical circumstance.

Applied technology has attributed a sense of real progress to medicine.^31^ The contemporary emphasis on ICTs in health care must be critically observed by society. Bioethics has the required competence to evaluate the effects of telemedicine on the integration of health sciences, health care professionals, patients/relatives, health institutions, and health care system.

The benefit of telemedicine should be considered more as a non-presential complementation of usual care rather than a replacement for face-to-face care. Telemedicine should be practiced with security and for a period relevant to the clinical circumstance (expiration dates proportional to the legitimate interests involved).^32,33^

An additional ethical aspect is that certain unavoidable perspectives of abuse of a technique should not adversely affect the beneficial use of the technique. Therefore, any ethical and legal considerations regarding the still young telemedicine, especially for application in a continental, multiethnic, and multicultural country like Brazil, cannot fail to recognize that it is difficult for a health care professional to define comprehensively and in depth his or her set of responsibilities, considering that the scope of telemedicine demands an A-to-Z range of intertwining requirements, decisions, and provisions regarding:

- involvement with fundamentals of current ethics, prudence, and zeal regarding complex issues like elderly care, comfort of vulnerable individuals, decrease in hospitalizations, and prompt guidance;
- impartial judgment about covering the patient’s real needs and constraint of secondary gains and conflicts of interest, including the potential for political (mis)use and power;
- sense of beneficence;
- avoidance of maleficence;
- commitment to the biological safety of the patient;
- respect for equity;
- definition about the complementary function of the “presential” and its substitute;
- awareness about the consequences of the “non-presential” on clinical reasoning;
- clarity about the range of use variations;
- training on roles, responsibilities, and skills in equipment management with continuous improvement;
- development of a friendly connection to the patients’ records;
- respect for the patient’s right to autonomy expressed through free, informed, renewable, and revocable consent;
- imperative appreciation of human values;
- critical appreciation of cost effectiveness;
- appreciation of the value of face-to-face relationships immediately or long before the online connection;
- creation of a mood of confidence despite the distance;
- conceptual and event-driven alerts about non-presential limitations;
- individual assessment of the level of competence for the required care at the moment;
- assessment of the completeness of the required information;
- concern with the continuity of the care provided;
- promotion of adherence to the recommended management;
- respect for professional confidentiality;
- “passport adjustments” related to the state geographical limitation of the physician’s registration in the medical council;
- continued research for reliable evidence of advantages and disadvantages;
- interface with consumerism in health care, including due and undue expectations about the possibility of immediate care;
- valuing the contribution of bioethics to the harmonization between classic, innovative, and novelty.

Therefore, in light of the existing ethical-normative bases of the current legislation and the bioethical principles that guide physician-patient relationships, we can establish the following guidelines for the use of telemedicine in cardiology:

Cardiologists should use caution, and prior to using telemedicine applied to cardiology (or simply telecardiology), they should maintain a fruitful relationship with their patients based on the Code of Medical Ethics.

The free and informed consent form is the document that obtains authorization from the patient for the use of telecardiology when the alternative of direct teleconsultation is considered.

Procedures for the remote monitoring of vital signs should be previously consented by the patient, with proper guidance and training regarding its use.

Medical companies providing telecardiology services must be registered with the Regional Medical Council (*Conselho Regional de Medicina*, CRM) and have a cardiologist as a technical manager, who will be in charge of overseeing the procedures performed, especially regarding the technological tools available to professionals.

Respect for the patients’ autonomy of will and protection of privacy regarding health data are the basis of telemedicine applied to cardiology.

### 1.6. Legislation and Regulation in Brazil

The Brazilian Internet Civil Framework (Federal Law No. 12.965, dated April 23, 2014)^34^ and the General Data Protection Law (LGPD; Federal Law No. 13.709, dated August 14 2018)^35^ are the main normative instruments with direct impact on telemedicine in Brazil, even though they are not specific for this purpose. The main authorities regulating telemedicine in Brazil are the Ministry of Health, the National Sanitary Surveillance Agency (Anvisa), the National Supplementary Health Agency (ANS), and the CFM.

#### 1.6.1. The Brazilian Internet Civil Framework

The Brazilian Internet Civil Framework (Decree 8.771, dated May 11, 2016)^34^ establishes the principles, guarantees, rights, and duties of users of the World Wide Web in Brazil.

The Brazilian Internet Civil Framework recognized legal relations in the virtual world and their effects on Brazilian order. In addition to establishing the neutrality of the web, it also excelled in safeguarding freedom of expression and privacy protection but failed to address important aspects related to personal data, leading to the development of the LGPD.34

#### 1.6.2. General Law Data Protection Law

Federal Law No. 13.709,^35^ dated August 14, 2018 (LGPD) deals with the processing of personal data, including digital information, by an individual or entity governed by public or private law, with the purpose of protecting the fundamental rights of freedom and privacy and the free development of the personality of the natural individual.

An important contribution of the LGPD^35^ is the clear definition of the concept of data:

- Personal data - information related to an identified or identifiable natural individual;
- Anonymized data - data related to a holder that cannot be identified by reasonable technical means available at the time of processing;
- Sensitive personal data - racial or ethnic origin; religious belief; political opinion; union affiliation or membership in religious, philosophical, or political organizations; health- or sex-related data; and genetic or biometric data, when linked to a natural individual.

According to the legislation, access to the Internet is essential to the exercise of citizenship, and inviolability of intimacy, privacy, and communications established through the Internet must be ensured to users.

Safety and confidentiality measures and procedures must be clearly informed by the service provider and should meet the standards set by regulations, respecting the right of confidentiality related to business privacy.

Regarding telemedicine, the need to deal with a large amount of sensitive data (patient registration, health complaints, prior and current disease history, test requests and results, diagnostic hypotheses, therapeutic plan, clinical follow-up, and opinions, among others) makes LGPD an object of significant interest.

In 2007, the Brazilian Ministry of Health established the National Telehealth Program to improve the quality of primary health care in the Unified Health System (SUS) and support the Family Health Strategy program. Ordinance 2.546, dated October 2011, expanded, redefined, and renamed the program to Brazilian National Telehealth Network Program, which governs the services of synchronous or asynchronous teleconsultation, telediagnosis, second formative opinion, and health tele-education.^36^

#### 1.6.3. Regulation of Telemedicine by the Federal Council of Medicine

According to CFM Resolution 1.643/2002,^37^telemedicine is the practice of medicine through the use of interactive audiovisual and data communication methodologies, with the objective of health care, education, and research. Additionally, the following relevant aspects should be highlighted:

- The services provided must have appropriate technological infrastructure and should comply with the CFM technical standards related to data storage, handling, transfer, confidentiality, and privacy, and must ensure professional secrecy.
- The professional responsibility for the care lies with the patient’s attending physician. Others involved in the process will be jointly liable to the extent to which they contribute to the eventual damage.
- Entities providing telemedicine services must be registered in the Entities Register of the Regional Council of Medicine of the state of their location along with a physician regularly registered in the Council assigned as a technical manager and a list of all physicians participating as staff members.

Since then, technological innovations and the democratization of Internet access have allowed several innovations that still lack proper regulation, such as:

- new means of physician-patient relationship;
- emergence of data and service agents and providers;
- discussion of a new format for the free and informed consent form under strict safety rules to guarantee information confidentiality and integrity.

This scenario prompted a need to update the regulation of telemedicine practice in Brazil. Based on that, the CFM issued Resolution 2.227/2018, which was later repealed. However, an update of the Resolution is urgently needed to provide legal security within the perspective of telemedicine emerging as a vector of health transformation.^38^

In this guideline, we adopt the denomination of the services offered within the scope of telemedicine, according to the Ministry of Health Ordinance 2.546, dated October 2011, and current CFM regulation.

### 1.7. Applicability in Brazil

In a country with continental proportions like Brazil, telemedicine represents a perspective to ensure the implementation of public policies conceived when the SUS was established, which have not been entirely fulfilled due to existing unassisted or remote areas lacking health care professionals, among other reasons. Thus, infrastructure conditions must be established to deliver available resources using health-related ICTs to these areas. To understand the applicability of telemedicine in Brazil, it is important to discuss concepts related to remote areas and to know the country’s medical demography.

#### 1.7.1. Concept of Urban and Rural Territories and Remote Area

The definition of territory goes beyond that of physical space since it generally has a strong relationship with the sociocultural context of the area. The division between urban and rural spaces is not abrupt; both have flexible boundaries and similar characteristics.^39^ Territorial occupation is evidently unequal in many regions, as it is also the access to goods and services offered in different forms of human settlements. In general, modes of transport and accessibility to urban and rural areas differ from one location to another, thus the importance of defining a classification for urban and rural concepts.^40^

According to the Organization for Economic Cooperation and Development (OECD), spaces are classified according to the population density, the proportion of the population living in large centers, and accessibility, defined as the commuting time between urban centers and rural areas. A rural area is classified as remote by the OECD when 50% of the local population requires at least 45 to 60 minutes of travel in motor vehicle to reach a center with a population of at least 50,000 inhabitants.^41^

In Brazil, the classification of occupied rural or urban spaces was established in 1938 by Decree No. 311/1938. The 2014 Territorial Base Manual, by the Brazilian Institute of Geography and Statistics (IBGE),^42^considers the access by national road or waterway network from rural areas to urban centers to classify rural areas according to their degree of proximity and access to large urban centers, creating a sense of isolation. The 2014 Transportation Logistics Map classified municipalities as adjacent or remote if the travel time from the municipal headquarters to a center of influence was longer or shorter, respectively, than the national average.

Table 1.1 shows the distribution of municipalities across the national territory based on the classification of isolation by IBGE.^43^

**Table 1.1:** Classification of isolation of Brazilian municipalities according to region and population^43^

Classification of isolation (IBGE)		Brazil	North	Northeast	Southeast	South	Midwest
Adjacent	Number of municipalities	5,126	277	1,683	1,637	1,180	349
	% of municipalities in relation to the large region	92.11	61.69	93.81	98.14	99.33	74.89
	Total population 2010 Census)	183,820,219	12,610,201	51,780,322	79,982,805	27,099,304	12,347,587
	% of the population	96.37	79.49	97.56	99.53	98.95	87.83
Remote	Number of municipalities	439	172	111	31	8	117
	% of municipalities in relation to the large region	7.89	38.31	6.19	1.86	0.67	25.11
	Total population (2010 Census)	6,927,512	3,254,253	1,293,560	381,605	287,587	1,710,507
	% of the population	3.63	20.51	2.44	0.47	1.05	12.17

Source: IBGE. Classification and Characterization of Rural and Urban Spaces in Brazil.^43^

**Figure 1.1:**
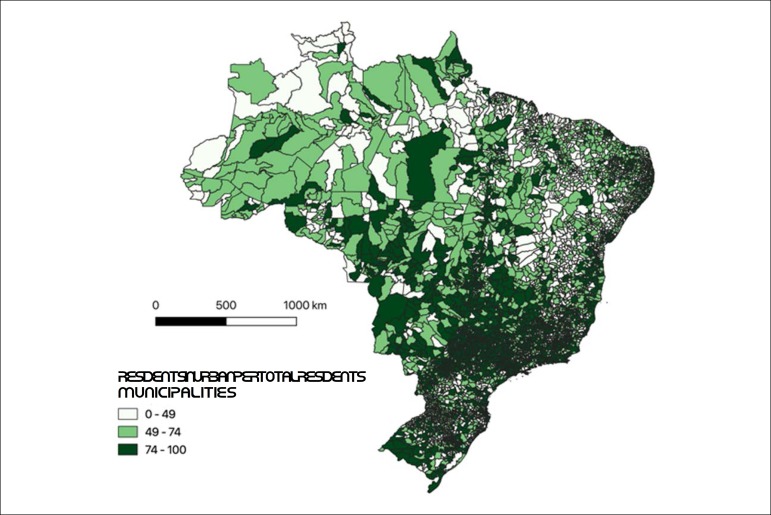
Proportion of urban population in Brazilian municipalities, 2010 census.^44^

Source: IBGE – 2010 census.^44^

More than 65% of the municipalities considered to be remote are located in the North and Midwest regions of the country. These two regions concentrate 5 million inhabitants or 72% of the country’s residents living in remote municipalities (almost 7 million individuals live in areas considered remote by the IBGE). Also in the North and Midwest regions, the population in remote municipalities represents 20% and 12% of the total population, respectively. Figure 1.1 shows the proportion of urban population in Brazilian municipalities.

#### 1.7.2. Medical Demography

The ratio of physicians per inhabitant in Brazil (2.1 physicians per thousand inhabitants) is significantly lower than the average ratio in OECD countries (3.4 physicians per thousand inhabitants). In addition to the absolute shortage of professionals, the country also has relative shortages due to large regional inequalities in the distribution of the existing medical workforce. Recent studies point out to a large concentration of medical professionals in the South and Southeast, with the proportion of specialists following this trend.^45^ Table 1.2 shows the distribution of physicians by country region, divided according to specialization as generalists, with some type of specialty (specialists), ratio per thousand inhabitants, and distribution of cardiologists by region and per inhabitant.

**Table 1.2:** Distribution of physicians by region of the country, disaggregated by specialization and region, grouped as generalists or specialists

Region	Physicians	Generalists	Specialists	Population	Cardiology	Cardiology/1,000 inhabs.	Physician/1,000 inhabs.
North	20,884	10,128	10,766	17,936,201	441	0.025	1.16
Northeast	80,623	34,461	46,162	57,254,159	2,534	0.044	1.41
Midwest	37,536	12,828	24,708	15,875,907	1,464	0.092	2.36
Southeast	244,304	91,124	153,180	86,949,714	8,383	0.096	2.81
South	68,430	20,948	47,482	29,644,948	2,694	0.091	2.31

Source: Scheffer M, Cassenote A, Guilloux AGA, Mioto BA, Mainardi GM. Medical Demography in Brazil 2018. São Paulo: FMUSP, CFM, Cremesp; 2018.^45^

*Population estimated by IBGE in 2017.

In the North and Northeast regions, some Federation units have a physician/inhabitant ratio below 1.00, like Pará and Maranhão, where the ratios are 0.97 and 0.87, respectively. The most recent Brazilian Medical Demographic Report (2018) also pointed to a significant inequality in the distribution of physicians between predominantly urban and rural municipalities, with a high concentration of the medical workforce in large urban centers.^45^

**Figure 1.2:**
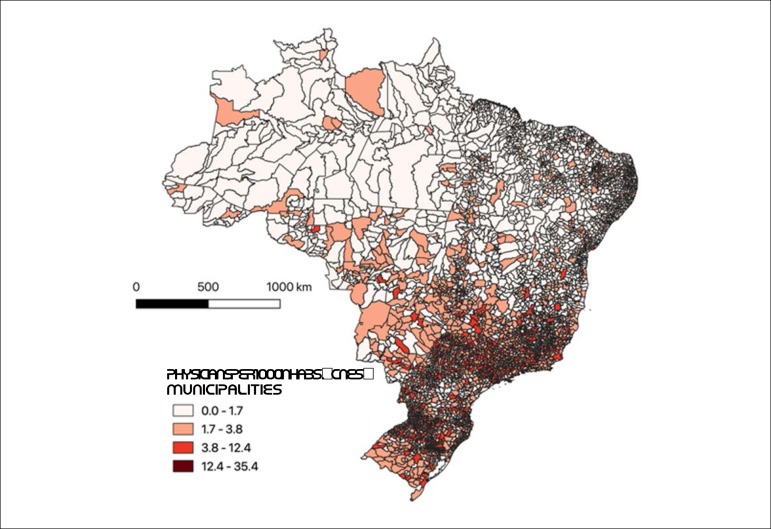
Distribution of physicians per thousand inhabitants in Brazilian municipalities – CNES, February 2019.^46^

Source: National Register of Health Facilities (CNES/DATASUS/MS).^46^

Data from the National Register of Health Establishments (*Cadastro Nacional de Estabelecimentos de Saúde,* CNES), provided by the Ministry of Health,^46^ show the same trend of concentration of medical professionals in the South and Southeast regions in February 2019, as seen in Figure 1.2.^46^

#### 1.7.3. eHealth Strategy

The International Telecommunication Union (ITU),^47^ an agency of the United Nations (UN), has been working in collaboration with the WHO to create a global environment for eHealth strategy implementation, especially in telemedicine.^47,48^

The eHealth strategy is particularly important in the control of chronic noncommunicable diseases like hypertension, diabetes, heart diseases, and age-related diseases. The implementation of eHealth and telemedicine has progressed substantially in recent years,^49^ but a recent systematic review on the cost effectiveness of eHealth implementation found shortage of studies and could not assess the impact of the strategy on health systems or social aspects, although most studies showed the strategy to be efficacious and cost effective.^49^

#### 1.7.4. Telecommunications and Data Infrastructure

**Figure 1.3:**
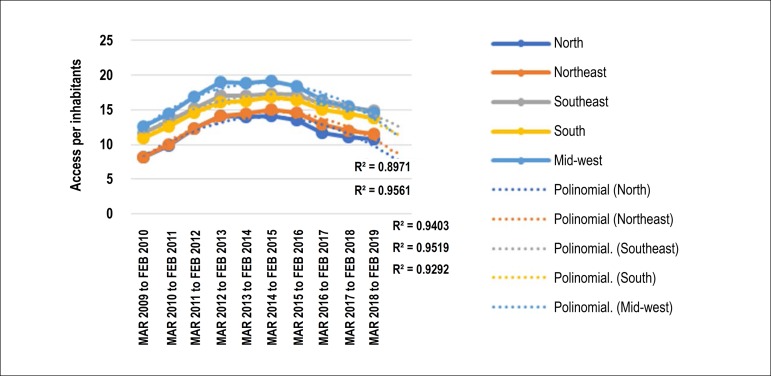
Density of access to mobile phones in Brazil and regions, March 2009 to 2019.^50^

Source: ANATEL.^50^

**Figure 1.4:**
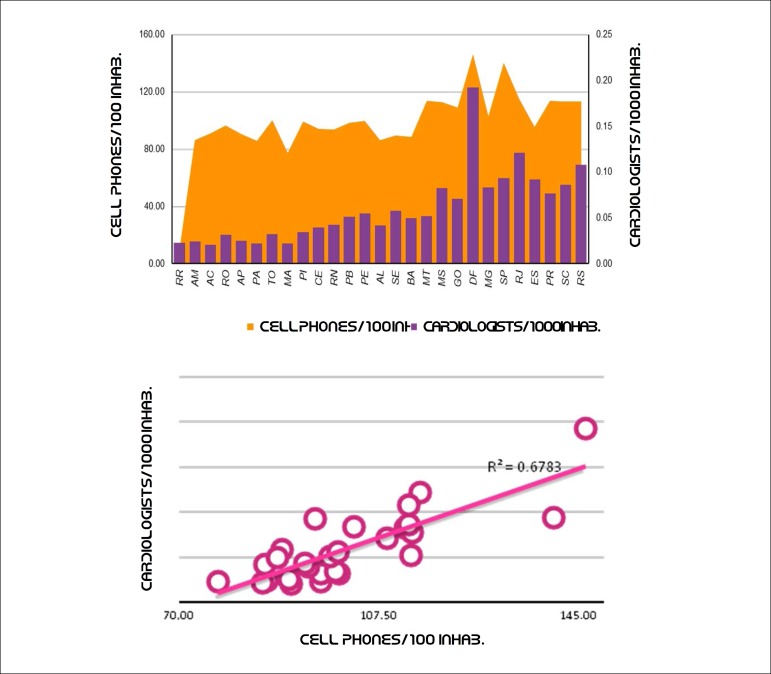
Distribution of cell phones and cardiologists, Brazil. a) Ratio cardiologists/1,000 inhabitants (2017), b) Density of cell phone density/100 inhabitants (2019).^51^

Source: Scheffer M, Cassenote A, Guilloux AGA, Mioto BA, Mainardi GM. Medical Demographics in Brazil 2018. São Paulo: FMUSP, CFM, Cremesp; 2018.^45^

Up to 95% of the world’s population is estimated to have access to mobile telephony; in Brazil, this coverage may exceed 98%. Access to mobile phone services has progressed remarkably in Brazil, and the use of mobile phone equipment per inhabitant has increased from 2009 to 2019,^50,51^followed by a downward trend since then (Figure 1.3). Figure 1.4 shows the distribution of cell phones per 100 inhabitants and the ratio between cardiologists and cell phones per 1,000 inhabitants in Brazil in 2018.

**Figure 1.5:**
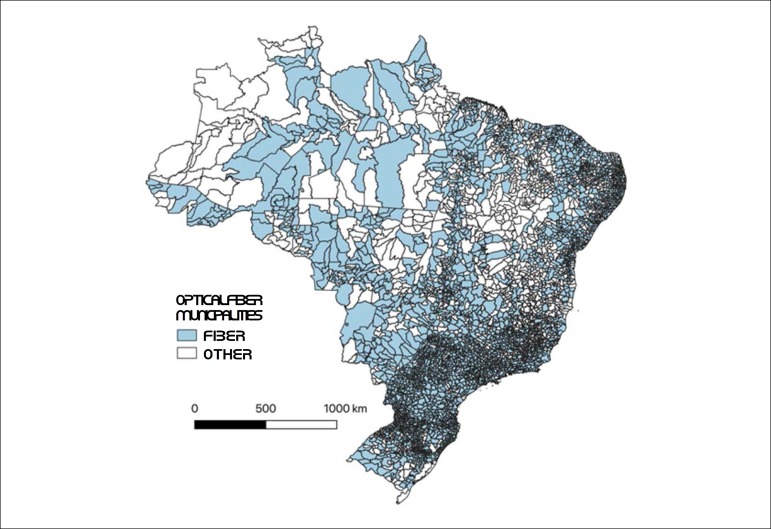
Municipalities with optical fiber backhaul and other technologies, February 2019.

Source: ANATEL.^50^

In terms of optical fiber coverage, the concentration is also greater in the Brazilian South and Southeast regions. Figure 1.5 shows the distribution of optical fiber backhaul in Brazilian municipalities. Backhaul is the portion of a hierarchical network (like cellular mobile communication networks) that is responsible for connecting the main network and the subnets. As shown in the map in figure 1.5, the concentration of optical fiber networks is lower in municipalities of the North region, which also concentrates the largest proportion of isolated municipalities.

Data from figures 1.4 and 1.5 show a trend of concentration of cardiologists in areas with a higher concentration of enabled mobile devices. The correlation coefficient of this relationship is 0.67, which indicates that the availability of cardiologists correlates highly with access to mobile phones. These data indicate a greater challenge to the implementation of telemedicine in remote areas, considering that the shortage of physicians follows the same distribution of the deficient telecommunications infrastructure in Brazil. A detailed analysis of the costs and benefits of this expansion should direct incentives to this area.

### 1.8. Artificial Intelligence

Artificial intelligence (AI) is a complex framework of sophisticated mathematical-computational models that allows the construction of algorithms to emulate various human tasks. AI encompasses an increasing number of subareas translating into different combined or complementary methodologies and approaches. Some examples include artificial neural networks (particularly deep learning models and convolutional networks), support vector machines, evolutionary algorithms, and natural language processing. The elaboration of analytical algorithms derived from large databases allows for interactive interpretation and apprehension, recognition of hidden patterns of combined information not obtained with traditional statistical methods, and assistance in more accurate decision making.

The availability of this huge amount of data and new analytical techniques - big data analytics - opens up new scientific possibilities and AI applications, such as machine learning and data mining, which are already widely applied in telecardiology to diagnose combinations of multiple modalities of images, biobanks, electronic cohorts, on-site and distance clinical monitoring sensors, electronic health records, genomes and other molecular techniques, among others.^52-54^

The implementation of these applications in clinical cardiology has grown exponentially^55^ and has prognostic features, like the use of an algorithm derived from magnetic resonance based on three-dimensional patterns of right ventricular systolic function to assess with high accuracy the outcomes in pulmonary arterial hypertension,^56^ identification of phenotypic patterns in heart failure with preserved ejection fraction and unfavorable prognosis confirmed by heterogeneous patterns of ventricular repolarization on electrocardiogram,^57^ prediction of cardiovascular risk in large cohorts,^58^ and prediction of urgent revascularization in emergency patients with chest pain,^59^ among others. However, AI studies are generally based on observational data from administrative databases or clinical records, which potentially have different types of biases and confounding factors.^54^

AI applications in telemedicine are promising but still very limited.^60^ In the area of telediagnosis, efforts for automated classification and diagnosis in electrocardiography and cardiovascular imaging^61^ are promising but still incipient. As for cardiovascular interventions, a recent review^62^ found 8 studies incorporating machine learning in a real-life research setting, of which only three were evaluated in a randomized controlled trial. Of the 8 interventions, 6 (75%) showed statistical significance (at a p level of 0.05) in health outcomes. Some of these interventions are directly related to telecardiology and assessed interventions for weight loss, stress control, smoking cessation, and personalized nutrition based on glycemic response. Most studies had small sample sizes and short duration, reflecting a need for investments and further studies exploring the potentialities in the area.

In a recent review, Topol^63^ highlighted the presuppositions that will guide the future of AI in medicine: the patient must be considered the center for the implementation of any new technology, the incorporation of these new technologies for diagnosis and treatment should occur after robust validation of their clinical effectiveness, the use of digital tools and decision algorithms by patients should be an option for those who feel empowered to do so, and interdisciplinary training must involve health care professionals, engineers, computer scientists, and bioinformaticians. These minimum conditions presuppose the steps to incorporate AI into clinical practice and minimize implementation challenges.

However, many aspects of health care practice will continue to depend on other dimensions, such as political, economic, and cultural ones, and on the ability of the health care professionals to interact with patients and community so that AI can truly benefit the patients, given that the issue of unequal access to health care is still critical in Brazil and will require large investments and reorganization of the health care system.^54^

Thus, potential strategies for incorporation and planning of implementation and adoption must be aligned with the possibility and challenges of offering cardiology care centered on the patient and the final value aggregated to the line of cardiology care. There is a need to identify the best technology to incorporate and define in which part of the medical work process such technology can add value to both the process and the patient’s health. Additionally, it is necessary to plan the incorporation and design the journey of digital transformation in cardiology to ensure a high technological level.

The incorporation of these technologies into clinical practice must, at first, involve rigorous evaluation of their performance and their ultimate value for the patient. This evaluation should respect and follow the current evaluation process of incorporation of new health technologies by the Ministry of Health, considering all aspects, norms, and regulations. The incorporation should also be based on scientific evidence on the generation of ultimate value to the patient’s health from the perspective of an individual exposed to technology.

It should be made clear that AI, once incorporated, works by increasing the professionals’ capabilities and never by replacing them, and that all civil and criminal responsibilities, as well as all responsibilities related to the patient and his or her health problem, remain with the attending physician.^64^

Training should be multiprofessional, interdisciplinary, and focused on building services dedicated to generating the ultimate value for the patient. The medical curriculum in the cardiology area should include contents related to technical knowledge, competence development, and use of AI techniques, while cardiology services should structure a continuous program for professional qualification and human resources training in managing incorporation, training, and adoption of new digital health technologies.

At present, there is no specific regulation on the use of AI in health care, although countries like Canada, United Kingdom, and the United States have begun the first phases of planning and implementing AI regulation in this area. Also, the European Union has published a document on the ethical aspects of AI in health care.^63^ The fast-paced digital transformation has led to reflections on how to balance the adoption of technology and emerging digital systems with ethical, moral, emotional, and social values, particularly values related to patients’ safety.

## 2. Uses and Application of Telemedicine in Cardiology

### 2.1 Telemedicine in Brazil

With the development of the Information Society in the late 20^th^ century as a result of globalization and widespread use of ICT, the emergence of organizational, social, political, and economic innovations of the society became pressing issues, requiring new ways to learn, teach, and work. The world began to worry about the principles of equal opportunity, participation, and integration so that everyone could access and benefit from the applications of the Information Society. In health care, telemedicine has made substantial progress worldwide, as it is classically viewed as a set of actions with great potential to improve access to health care services and to care quality and effectiveness at a lower cost.^65^

As a mark of the new millennium, we highlight the aging of the population, the increase in chronic noncommunicable diseases, and the consequent need to provide health care services for a longer time, which increases health-related costs. Therefore, it is essential to incorporate innovative, efficient, and effective solutions like telemedicine and biotechnology to promote universality and comprehensiveness in health care.

Several actions in telemedicine are currently present in all continents and must be planned according to local needs in order to be successful. According to Bashshur et al.,^66^ the success of these actions depends on three pillars: access, quality, and cost.^66^ In developed countries, telemedicine is an alternative to traditional methods and is already present as an option to supplementary health or to address gaps in the health care system, but always aiming at integral care. In developing countries, access is the main pillar, since telemedicine can be the only option in regions where traditional specialized care is not available.

In Brazil, the systematic development of telemedicine and telehealth in the public health system started in 2006, with investments from the Ministry of Health, State Health Secretariats, and Municipal Health Secretariats. The main objective was to support primary care, particularly the Family Health Strategy in remote municipalities, through teleconsulting, telediagnosis, and tele-education. If applied on a large scale, these strategies could decrease the referral of patients to large centers and consequently improve the population’s access to specialized care and reduce health care costs.^67^ Therefore, telemedicine in the Brazilian public system has been anchored from the outset in the basic principles of universality, equity, and integrality of the SUS. Based on the universality principle, health is everyone’s right, and it is up to the state to ensure it. Equity targets the reduction of inequalities or increased investment in areas where it’s most needed. Integrality considers the individual as a whole to meet all his or her needs.^68^

Telecardiology, one of the most developed domains in telemedicine, has multiple actions in promoting health, disease prevention, diagnosis, treatment, and rehabilitation with an impact on the quality of life. It can be considered an important ally of the public, supplementary, or private health care system in promoting comprehensive and high-quality health care.

### 2.2. In Primary Care

Primary Health Care (PHC) involves integrated and multidisciplinary care and is the foundation to achieve universal health, according to the PAHO, which also advocates for other health determinants like education, food, housing, financial protection, clean water, and safe environments.^69^ To achieve universal health, health care systems must be transformed, especially by making PHC efficient, integrated, and organized, placing the patient at the center of the system. The PAHO also estimates that about one third of the population in the Americas has no access to health care and that 800,000 additional health care professionals would be necessary to meet the needs in the region.

Telemedicine plays an important role in the qualification of the PHC, with clinical, human, organizational, educational, administrative, technical, and social benefits.^70^ The application of telemedicine to support PHC brings benefits to the population served, including (i) improved access to specialized services, (ii) increased solvability to the basic level, (iii) decreased number of patients referred to other municipalities for specialized care, (iv) more qualified referrals and faster hospitalization decisions, (v) better training of local health care professionals, with consequently better qualified clinical care, (vi) reduced time to diagnosis, with decreased risk of complications, (vii) diagnosis of diseases at earlier stages, (viii) cost and time savings for the patient, (ix) improved quality of life, (x) fewer hospitalizations and visits to emergency units, (xi) improved clinical care continuum, (xii) reduced risk factors and complications from chronic diseases, and (xiii) savings for the health care system.^70-74^

#### 2.2.1. Applications in Health Promotion and Prevention

In cardiology, health promoting actions and primary and secondary prevention of cardiovascular diseases translate into significant cost savings by reducing specialized consultations, hospitalizations for clinical complications, and admissions to the emergency room.^71^ Telemedicine can be useful in controlling risk factors for coronary artery disease; improving blood pressure control;^75-78^ reducing glycosylated hemoglobin levels in patients with diabetes mellitus;^79-81^ improving lipid profile;^82,83^ reducing weight, body mass index (BMI), or waist circumference in obese individuals;^77,84-86^ and increasing the success of smoking cessation programs.^87^

Several modalities of telemedicine can be applied in this regard, including cell phone text/audio messaging systems, which have positive results in improving medication adherence, changing eating habits, and increasing physical activity among patients with hypertension, diabetes, and obesity, or after acute myocardial infarction (AMI).^86,88^ 24-hour monitoring systems on cell phones or monitoring center services have become more frequent due to the development of specialized equipment with direct communication with telemedicine systems such as stethoscopes, scales, thermometers, digital devices, blood pressure equipment, remote monitoring of vital signs and implantable electronic devices.^89,90^ Simple watches have been transformed into monitoring systems equipped with technology to report heart rate, stress level (skin humidity and temperature), optical BP, and physical activity, among other parameters.^91-92^ Several applications are available to guide the health care team and/or patients, including applications focused on self-care.^89^

#### 2.2.2. Decision Support Systems

Clinical decision support systems (DSSs) provide knowledge and information from individual patients to physicians and other health care professionals, or to the patients themselves, to improve care quality and clinical outcomes. These systems are recommended by the Community Preventive Services Task Force (CPSTF) in the prevention of cardiovascular diseases despite being based on moderate- to poor-quality evidence.93 Applications that have shown benefits include those improving screening for cardiovascular risk factors, prescription of aspirin for primary prevention, and counseling on healthy diet, physical activity, and smoking cessation.94 Due to that, these applications may have wide applicability in PHC. Still, they have shown no evidence of reducing emergency visits, hospitalizations, or cardiovascular events, although additional studies are still needed. A study reported a lower mortality rate with an educational strategy for health care professionals associated with DSS alerts compared with educational strategy alone.^95^

DSSs have been evaluated in pilot studies in Brazilian Basic Health Units (*Unidades Básicas de Saúde,* UBS) in multifaceted interventions. This tool was proven feasible in PHC in patients with hypertension and diabetes and for cardiovascular risk management, with good satisfaction reported by the professionals and perceived ease of use,^96,97^ although the number of questionnaire fields filled in by the professionals was low.^96^ This may be related to the incipient implantation of electronic medical records in UBSs, generating duplicate work, a factor that was inversely related to the successful implementation of the DSS.^98^ New initiatives are underway to assess the impact on clinical outcomes of the control of patients with hypertension and diabetes in the Mucuri Valley, in Minas Gerais, and the management of patients on warfarin, still the most widely used anticoagulant in the SUS.

#### 2.2.3. Teleconsulting

Teleconsulting systems have great applicability in PHC in terms of supporting health care professionals in remote areas and qualifying and reducing the time to diagnosis and treatment. As a tool with the important potential of increasing PHC solvability, teleconsulting should be incorporated into the care process in health care units as an integral part of the regulatory process of the municipality. This is an efficient way to reduce the long wait for in-person consultations with a cardiologist. Although teleconsultation has been extensively studied in our country,^99^ only a few studies have evaluated in our population the impact of teleconsultation on traditional health outcomes, like risk and mortality. A systematic review by Liddy et al.^100^ reported that teleconsulting systems were highly accepted by patients and health care professionals and improved access to specialized care. A randomized trial in cardiology assessed adverse events (including death, AMI, urgent or emergent cardiac catheterization and/or angioplasty, and emergency room visits) in patients referred to teleconsultation versus patients receiving a traditional referral. The group referred to teleconsultation was more likely to have an appointment with the cardiologist and had fewer visits to the emergency department.^101^

#### 2.2.4. Teleregulation

The demand for specialized care has been growing worldwide and surpassing the supply of services while meeting limited access to specialists and long waiting times.^101^ Interventions in telehealth, particularly involving teleconsultation for regulation, have shown a great impact in reducing waiting time with the qualification of access to specialists, avoiding unnecessary referrals, and at a lower cost. In Brazil, the experience of teleconsultation for regulation (or teleregulation) has also reduced the waiting time for specialized consultation, qualifying the access and optimizing the use of resources, in addition to providing users with greater comfort and lower impact on their routine.^102^-^104^ Teleregulation also enables the classification of the risk of the demand for specialized care. Protocols to guide health care have been established, and the final decision regarding referral is defined along with the attending and teleconsulting physicians. In addition to the mentioned gains from the user’s perspective, there is the process of continuing education and professional qualification, increasing solvability in primary care.^101-104^

#### 2.2.5. Telediagnosis

Tele-electrocardiography, the most common activity in telecardiology, is a simple and low-cost technology for easy transfer of a small file using an Internet connection with limited bandwidth. Thus, it can be easily incorporated into the PHC routine for its great applicability and suitability for the infrastructure of PHC facilities in remote and poor areas.^105,106^ Tele-electrocardiography is widespread in both public and private settings in Brazil, with several companies in the country delivering reports around the clock. In 2017, the Ministry of Health launched the National Offer of Telediagnosis Project (*Projeto Oferta Nacional de Telediagnóstico,* ONTD) to expand the diagnostic services of tests conducted remotely in the most deprived areas of the country. Tele-electrocardiography was the first modality of telediagnosis offered nationwide by a telehealth team specialized in telecardiology. This project is an innovation in the management of a large-scale national telemedicine project model, and the good results obtained have shown its easy applicability and suitability for remote areas.^107^ The application of AI to the large databases of diagnostic tests improves the ease of the process of reporting and increases the accuracy of the tests.^61,108^

In telecardiology, tele-echocardiography is a promising strategy for rationalization of the access to complementary propaedeutics, early diagnosis, prioritization of referrals, and organization of waiting lists. Initial evidence of tele-echocardiography application derive from population-based screening studies, for example, a study conducted in rural India, where more than 1,000 echocardiograms were performed in about 11 hours and transferred to a cloud computing system for expert analysis using telemedicine.^109^ The strategy proved feasible and showed good agreement between preliminary field diagnoses and the reports by experts (k = 0.85), and an alarming 16% rate of major abnormalities (including 32.9% of valvular defects). Even in high-income regions like the UK, evidence has shown echocardiographic screening in primary care by nonspecialists to be an attractive strategy, with clinically significant (moderate to severe) valvular disease observed in 6.4% of the asymptomatic population aged ≥ 65 years and associated with socioeconomic factors.^110^ The strategy may be especially useful in Brazil, which has a presumably high burden of undiagnosed cardiovascular disease and limitations in the provision of specialized tests, including conventional echocardiography.

The tele-echocardiography strategy was first tested in Brazil in a program for screening of rheumatic heart disease (the Rheumatic Valvular Disease Screening Program study; *Programa de RastreamentO da VAlvopatia Reumática* - PROVAR). The study established, at a research level, a routine for the acquisition of simplified imaging protocols using portable and ultraportable devices by non-physicians (nurses and technologists), which were uploaded to dedicated cloud computing systems for storage and remote expert interpretation.^111,112^ In addition to remote diagnosis, telemedicine was also used to train health care professionals on basic echocardiographic principles through interactive online modules. After training, health care professionals with different backgrounds were able to diagnose rheumatic heart disease with accuracy.^111^ The project reported a high prevalence of subclinical rheumatic heart disease (4.2%), which is quite significant considering the current impact of the disease on public health.^113^

A similar strategy was subsequently applied in primary care. Professionals (physicians, nurses, nurse technicians) from health care centers located in low-income regions of metropolitan areas of Belo Horizonte and Montes Claros received online and in-person training for the acquisition of a simplified echocardiographic protocol with ultraportable devices and support by the project’s field team. Echocardiography was performed in asymptomatic individuals of three age groups (17-20, 35-40, and 60-65 years) as a screening method and in patients on a waiting list to undergo echocardiography or who had this test requested by the family health care team. The results showed that a) the strategy is feasible for the conditions found in Brazil and has the potential to be expanded to other scenarios; b) the prevalence of echocardiographic abnormalities in asymptomatic populations was high (above 20%) in general; c) among patients on a waiting list for echocardiography, more than 50% had no significant abnormalities on screening echocardiography; and d) the correlation with conventional echocardiography was satisfactory.^112^ A prediction score was developed from these findings, incorporating clinical data and variables from the simplified echocardiogram.^114^Thus, tele-echocardiography may be a strategy for early diagnosis but is mainly an instrument to prioritize and organize waiting lists in health care systems with limited availability of tests and specialized consultations. However, the incorporation of this model into Brazilian health policies depends on broad regulatory discussions involving authorities, professional councils, and medical societies - especially regarding simplified image acquisition by non-physicians.

The adoption of tele-echocardiography (Table 2.1) for the care of disadvantaged remote communities has potential advantages, but this method still lacks robust scientific validation with prospective controlled studies confirming its benefits to patients’ health and cost-effectiveness, among other challenges.

**Table 2.1:** Potential advantages and challenges for the adoption of tele-echocardiography in Brazil

Advantages	Challenges
Allow access to the method at remote locations	Lack of standardization of the components of tele-echocardiography
Optimization of clinical outcomes	Absence of scientific evidence confirming the impact on clinical outcomes
Reduction in the cost of transporting human resources to geographically distant areas	Absence of scientific evidence confirming cost-effectiveness; questions about reimbursement and system costs
Reduction in the cost of transporting patients to tertiary centers	Uncertainty about adherence by local health care professionals
Reduction in the number of unnecessary echocardiograms	Veto of the Brazilian legislation to the work of non-medical operators (sonographers)
	Lack of guidelines for training of operators
	Medical-legal uncertainties
	Legislative issues related to licensing, data storage, privacy, and confidentiality

#### 2.2.6. Tele-Education

Remote educational activities in cardiology for health care professionals, offering courses, lectures, and learning tools on clinical issues and care management, have the added benefit of improving the quality of care. Educational activities for patients should be encouraged for their health empowerment.

In remote municipalities with small populations, PHC is often the only level of local health care, while their health care units receive patients with acute cardiovascular diseases. Thus, telecardiology in PHC not only should qualify the care of chronic diseases but should also support urgent care for ischemic diseases and arrhythmias.

Due to the myriad of applications of simple telemedicine tools, cardiology can be considered one of the specialties most sensitive to the use of ICTs. The triad teleconsulting, telediagnosis, and tele-education integrally applied to PHC and associated with tools like DSS can make a difference in the quality of care for cardiovascular diseases, especially hypertension, atrial fibrillation, heart failure, and AMI. Finally, teleregulation can offer support to PHC, in terms of solvability at this level, improving access to specialty care.

### 2.7. In Specialized Care

#### 2.7.1. Heart Failure

Extensive literature has examined the use of telemedicine strategies to monitor patients with heart failure with the objective of reducing hospitalizations associated with increased morbidity, mortality, and costs and improving patients’ adherence and participation. The interventions range from the application of traditional technologies like structured telephone support, telemonitoring using innovative technologies with implantable or wearable devices, DSS, and machine learning to predict complications.^115,116,117^ The results are variable, but most demonstrate benefits. However, the use of these strategies in clinical practice is still very limited due to regulatory, logistics, and financial issues.^118^

Telemonitoring may be invasive or noninvasive. Sensors are tools capable of detecting, recording, and responding to specific information, *e.g.,* patients’ vital signs, and are increasingly embedded in smartphones and other mobile devices. Sensor logging can generate large data sets that may be transmitted in real time to health care professionals.^119^ Since multiprofessional intervention programs often have geographical, economic, and bureaucratic barriers, telemonitoring can be a solution to promote care for patients with heart failure.^115^

Evidence about structured telephone support and noninvasive telemonitoring in patients with heart failure has been summarized in a Cochrane systematic review of 41 studies. Structured telephone support reduced all-cause mortality (RR 0.87, 95%CI 0.77-0.98; n = 9,222) and heart failure-related hospitalizations (RR 0.85, 95%CI 0.77-0.93; n = 7,030), both with moderate-quality evidence. Telemonitoring reduced all-cause mortality (RR 0.80, 95%CI 0.68-0.94; n = 3,740) and heart failure-related hospitalizations (RR 0.71, 95%CI 0.60-0.83; n = 2,148), both with moderate-quality evidence.^119^

Another meta-analysis^120^ of 26 studies with 2,506 patients undergoing telemonitoring (including the transmission of vital signs) observed a time-dependent effect. Short-term follow-up (up to 180 days) had better results for hard outcomes (like mortality), which were not maintained during longer follow-up (1 year). Regardless of the follow-up duration, the strategy was unable to reduce hospitalization. An increase in emergency visits in the telemonitoring group raises the question of how an intervention that does not reduce hospitalization could reduce the mortality rate. Perhaps the early detection of signs of decompensation encourages a more frequent search for care and prompt treatment with diuretics and vasodilators without requiring intensive therapy.

In the publication of one of its Clinical Protocols and Guidelines on Heart Failure,^121^the Ministry of Health analyzed several studies evaluating the benefits of telemonitoring based on telephone follow-up, recommending for health care services to consider follow-up using telephone support for patients with New York Heart Association (NYHA) functional class III to IV heart failure after hospital discharge. The analysis showed an 18% reduction in overall mortality with remote monitoring compared with usual care (RR 0.82, 95%CI 0.73-0.93). Telemonitoring also reduced by 23% (RR 0.77, 95%CI 0.68-0.88) the risk of hospitalization due to heart failure. Of note, this recommendation should be directed to patients with the potential of most benefits. There is no consensus on the intensity and frequency of monitoring, but they should be performed focused on clinical and educational guidance.

Evidence regarding the duration of hospital stay is more fragile and controversial. Of seven studies on structured telephone support and nine on telemonitoring, only one on each type of intervention reported significantly decreased hospital stay. Additionally, nine of 11 studies on structured telephone support and five of 11 studies on telemonitoring reported significant improvements in quality of life. Three of nine studies on structured telephone support and one of six studies on telemonitoring reported reductions in cost, while two studies on telemonitoring reported increased costs due to expenses related to the intervention and increased medical management. Seven of the nine studies that assessed heart failure knowledge and self-care noted significant improvements. Despite acceptance by 76-97% of the participants, decreased adherence to the intervention over time can be challenging, and was reported in the review at 55.1-65.8% with structured telephone support and 75-98.5% with telemonitoring.^119^

Machine learning techniques can be potentially valuable in remote monitoring of patients at high risk of heart failure. Individual characteristics of these patients obtained from the analysis of a large number of electronic medical records may help identify those at greatest risk of unfavorable outcomes who could benefit from individualized medical treatment.^122^ The Seattle Heart Failure Model (SHFM), for example, is a machine learning framework for the calculation of mortality risk in heart failure. The model considers various clinical aspects obtained from electronic medical records to predict the prognosis of the disease and incorporates the potential impact of therapies on patients’ outcomes. This DSS was shown to be potentially useful in identifying patients with heart failure at higher risk of unfavorable outcomes, but met implementation barriers, as it was time consuming, expensive, required familiarity of the physician with computers, and failed to take into account other clinical variables that were not included in the collected data.^123^

Evidence of the benefits of telemonitoring in heart failure has been recently confirmed with the publication of the Telemedical Interventional Management in Heart Failure II (TIM-HF2) study. This was a prospective, randomized, multicenter clinical trial including 1,571 patients with NYHA class II or III heart failure hospitalized due to heart failure within 12 months before the randomization and with an ejection fraction of 45% or lower. The patients were then randomized to remote management or usual care and followed up for up to 393 days.^124^

The percentage of days lost due to unplanned cardiovascular hospitalizations and death from all causes was 4.88% in the remote patient management group and 6.64% in the usual care group (p = 0.04). Patients assigned to remote management lost an average of 17.8 days/year compared with 24.2 days/year among patients assigned to usual care. The hazard ratio (HR) for all-cause mortality was 0.70 (95%CI 0.50-0.96; p = 0.0280) in favor of the teleconsultation group, but cardiovascular mortality was not significantly different between both groups (HR 0.671, 95%CI 0.45-1.01; p = 0.0560).^124^

New devices to monitor intracardiac pressure present the most compelling evidence for the application of telemonitoring and use more advanced technologies. CardioMEMS is a device that is percutaneously implanted in the pulmonary artery to transmit central pressure values to a platform. When the pressure levels of the pulmonary artery rise above a certain threshold, the physician receives an alert and a statement indicating congestion or low output. Other devices for implantation in the right ventricle are being used experimentally. The study CHAMPION (CardioMEMS Heart Sensor Allows Monitoring of Pressure to Improve Outcomes in NYHA Class III Heart Failure Patients)^125^ evaluated patients with NYHA functional class III heart failure across 64 centers in the US. The patients were randomized by a centralized electronic system to a group of management by CardioMEMS or to a control group.

In the monitoring group, the physicians used daily data from pulmonary artery pressure measurements to guide treatment. After a follow-up of 15 months, the monitoring group had a 37% reduction in hospitalizations related to heart failure compared with the control group. The long-term follow-up of this study, in which the control group was switched to receive pulmonary pressure monitoring, showed that these results remained significant and clinically relevant over time.^126^

#### 2.7.2. In Hypertension

Telemonitoring strategies can also be applied for BP control, but they overlap the self- monitoring approach. In the TASMINH4 trial, 1,182 patients were randomized (1:1:1) to general titration of antihypertensive medication based on clinical readings by a generalist (usual care group), self-monitoring (self-monitoring group), or self-monitoring along with telemonitoring (telemonitoring group). The study found that the use of BP self-monitoring to titrate antihypertensive therapy in poorly controlled hypertension in primary care resulted in lower systolic BP without increasing the workload of the clinical team. After 1 year, patients who had the medications adjusted based on self-monitoring with or without telemonitoring had significantly lower systolic BP than those who had the treatment adjusted based on BP measured during consultations. The BP values in the telemonitoring group that received medication titration became lower faster (at 6 months) than in those in the self-monitoring group, an effect that is likely to further reduce cardiovascular events and improve long-term control.^127,128^

Several studies also show that strategies for hypertension telemonitoring involving a clinical pharmacist lead to a beneficial impact on BP control in the short and medium term. Margolis et al.^129^ evaluated the durability of the effect of such intervention after a follow-up of 54 months in a randomized cluster study among 16 primary care clinics and 450 patients (228 receiving telemonitoring and 222 on usual care). Intensive intervention based on telemonitoring maintained the effects for up to 24 months (12 months after the end of the intervention), but lost efficacy in the long term.^129^

A prospective observational cohort study monitored the BP levels before and after an educational intervention and introduction to home BP monitoring (HBPM). In the intervention group, 484 patients were instructed to track their BP levels using a smartphone three to seven times a week. The mean BP levels improved from 42% to 67% among patients on HBPM compared with 59% to 67% among controls (p < 0.01).^130^

The INTERACT study was a randomized clinical trial in which 303 patients using BP and/or lipid-lowering medications were randomized to receive or not receive text messages. The group that received text messages improved medication adherence at 6 months compared with the group that did not receive messages. The overall improvement in medication adherence was 16%.^129,131^

A Cochrane systematic review^132^ sought to establish the effectiveness of mobile phone-based interventions in improving adherence to medications prescribed for primary prevention of cardiovascular disease in adults. The participants in the trials were recruited from community-based primary care or outpatient clinics in high-income (Canada, Spain) and upper- to middle-income countries (South Africa, China), but the interventions varied widely. One trial evaluated an intervention focused on adherence to BP medication delivered exclusively by text messaging, while another trial involved BP monitoring combined with feedback delivered via smartphone. The authors considered the body of evidence for the effect of cell phone-based interventions on objective outcomes (BP and cholesterol) having a low quality. Of two studies that evaluated medication adherence along with other lifestyle modifications, one reported a small beneficial effect on lowering low-density lipoprotein cholesterol while the other found no benefit. Another trial (1,372 participants) on an intervention based on text messaging showed a small reduction in systolic BP in a group that delivered information-only text messages, but uncertain evidence of benefit in a second intervention group that provided additional interactivity. One study examined the effect of BP monitoring combined with smartphone text messaging and reported moderate intervention benefits to systolic and diastolic BP. There was conflicting evidence from trials targeting medication adherence along with lifestyle advice using multicomponent interventions. Another study found large benefits on BP levels, while another study showed no such effect. The authors of this Cochrane review concluded that there is low-quality evidence related to the effects of interventions delivered via mobile phone in increasing adherence to medications prescribed for primary prevention. The conclusion based on this review is that there is current uncertainty about the effectiveness of such interventions.

#### 2.7.3. Emergency Services

Brazil has a geographically distributed health care system in which UBSs, emergency care units (ECUs), secondary hospitals, and ambulances are scattered across the country, often at remote locations. Specialized centers are located in advanced care units, like tertiary hospitals, located in major cities. In this context, telemedicine tools can improve emergency management.^133^

Telemedicine has different applications in emergency services, ranging from electrocardiographic transmissions associated or not with synchronous teleconsultations to assist in the early diagnosis and management of cases of acute coronary syndrome (ACS); clinical DSSs to help with the diagnosis, management, and prediction of cardiac complications in patients with ACS;^133^ transmission of bedside ultrasonographic images before hospital admissions;^134^ and image transmission and support in the diagnosis and management of patients with acute stroke.^135^ The use of DSSs could increase the adherence to guideline recommendations in the management of patients with ACS, but evidence on its impact on clinical outcomes in this context is still limited.^135^

#### 2.7.4. In Systems of Care for Acute Myocardial Infarction

Systems of care for AMI integrate preadmission services, hospitals, and hemodynamic services comprising the care of patients with AMI in a given region in order to optimize the management of clinically suspected patients. The proposal of these systems is to delineate the patients’ care flow involving early diagnosis, preadmission care, initial treatment, use of thrombolytic agents, referral to a specialized hospital, and post-event follow-up. They target high-quality, effective, safe care for patients with AMI by optimizing resources and reducing disparities in their access to care.^133,136^

Telemedicine services may play a crucial role in AMI systems of care, as they facilitate the communication between a physician in an emergency unit, low-complexity hospital, or pre-hospital admission with cardiologists at the hub or hospital with a hemodynamic center that will receive the patient. Cardiologists can assist in (i) analyzing and interpreting electrocardiograms for accurate and early diagnosis of ST-segment elevation AMI,^132,137^ (ii) guiding the best course of action, including the administration or not of thrombolytic agents and other medications, through synchronous teleconsultations, *i.e.*, real-time communication between the on-site professional and the remote specialist,^137,138^ and (iii) monitoring the patient’s clinical condition through telemonitoring, with synchronous data transmission.^137^

The incorporation of telemedicine strategies in systems of care for AMI is a worldwide trend. A recent meta-analysis including studies conducted in Europe (11), North America (8), South America (5), Asia (9), and Australia (2), with a total of 16,960 patients, found consistent moderate-quality evidence that telemedicine strategies associated with usual care in this context reduce in-hospital mortality by 37% (RR 0.63, 95%CI 0.55-0.72), with a number needed to treat (NNT) of 29 (95%CI 23-40) when compared with usual care without telemedicine. The study also found poor quality evidence that this intervention can reduce door-to-balloon time (mean difference 28 minutes, 95%CI -35 to -20 minutes) and 30-day (RR 0.62, 95%CI 0.43-0.85) and long-term (RR 0.61 95%CI 0.40-0.92) mortality.^138^

In Brazil, Belo Horizonte, Campinas, Salvador, São Paulo, and the Northern Region of Minas Gerais (encompassing 89 municipalities) have published initiatives in this area.^137,139-143^ Decreased system delays and increased reperfusion rates have been observed in cases of ST-segment elevation AMI, with evidence of reduced hospital mortality.^139,143,144^

A typical telemedicine system comprises a specialized center (hub) and multiple remote care units distributed within a geographic region (spoke centers) connected bidirectionally by a communication channel. The specialized center may be a referral hospital in cardiology, the operation center of an Emergency Mobile Care Service (*Serviço de Atendimento Móvel de Urgência*, SAMU), or a telemedicine center. Some systems of care for AMI comprise more than one specialized center, each with specific remote units for regional coverage.^145^ The 2015 Telemecardiology Guideline for the Care of Patients with Acute Coronary Syndrome and Other Heart Diseases details models of care using telemedicine systems for the care of patients with ACS.^133^

#### 2.7.5. In Controlling the Use of Anticoagulants

The strategy of self-management of anticoagulants has been associated with a significantly lower risk of ischemic stroke and all-cause mortality compared with direct treatment with oral anticoagulants, while no significant differences were observed for major bleeding and mortality. However, decreased surveillance is a potential problem for the detection of patients who are unable to take care of their own treatment. A structured education program is required for patients and/or caregivers and for involved professionals in health care and quality control.^146-148^

#### 2.7.6. Cardiac Rehabilitation

Guidelines recommend that patients should undergo cardiac rehabilitation after AMI, percutaneous coronary intervention (PCI), or myocardial revascularization. However, rehabilitation is still underused, with participation of only 14-31% of all eligible patients. Patients’ inability to attend the sessions and costs are important barriers.^149^ Telehealth interventions using ICTs to enable remote rehabilitation programs can overcome common barriers to rehabilitation access while preserving clinical supervision and prescription of individualized exercise.^150^

In a systematic review of 11 studies, the types of intervention were variable and included the use of mobile or computer applications, biosensors, and interventions delivered by landline phone lines. The interventions involved prescription and/or monitoring of the participants’ performance and adherence. All interventions included feedback, education, psychosocial support, and/or behavioral changes via landline phone communications, mobile messaging, e-mail, website use, online tutorial, or online chat.^151^

The level of physical activity was higher in the intervention group compared with the usual care group. Compared with face-to-face rehabilitation, the intervention with telehealth was more effective in improving the level of physical activity, exercise adherence, diastolic BP, and LDL-cholesterol, with poor- to moderate-quality evidence. Telehealth rehabilitation was similar to face-to-face rehabilitation in maximal aerobic exercise capacity and other modifiable cardiovascular risk factors.^151^

The Telehab III study was a prospective, randomized, multicenter controlled trial with patients undergoing cardiac rehabilitation. In all, 140 patients were randomized to a conventional rehabilitation group or a 24-week Internet-based telerehabilitation group associated with conventional rehabilitation. The additional telerehabilitation program showed improvement in physical fitness and quality of life and induced persistent health benefits.^152^

A clinical trial conducted in China randomized 98 patients with NYHA I-III functional class heart failure to an 8-week home-based teleconsultation exercise training program or usual outpatient follow-up. Statistically significant improvements were observed in the experimental group in terms of quality of life and result in the 6-minute walking distance test compared with the control group. These results confirm that physical training via teleconsultation is an effective alternative method for cardiac rehabilitation.^153^

The noninferiority, randomized controlled trial REMOTE-CR tested the effects and costs of cardiac rehabilitation by real-time teleconsultation in 162 patients with heart failure and demonstrated this to be a cost-effective alternative that can enhance the scope of rehabilitation.^154^

Home-based cardiac rehabilitation is an alternative to increase patients’ engagement in the program by presenting greater flexibility and activity options, offering choices based on the patients’ values and preferences, and allowing the implementation according to the patients’ daily routine.^155^ The association of cardiac rehabilitation with conventional rehabilitation has been shown to be more effective and efficient compared with a conventional rehabilitation program alone, as it reduced the rates of readmission due to cardiovascular causes and improved quality of life during the study period.^156^

#### 2.7.7. Remote Monitoring by Implantable Devices

Pacemaker telemonitoring has not significantly improved quality of life and number of cardiovascular events, but provided early detection and treatment of events, reducing hospital admissions and visits (routine and emergency) at lower costs compared with hospital follow-up.^157^

Implantable cardiac defibrillators (ICDs) or resynchronization defibrillators are another type of implantable monitoring systems. Some of these devices may be equipped with software for multiparametric monitoring of, for example, thoracic impedance and right ventricular filling pressure with measurements captured by a right ventricular lead. A groundbreaking study published in 2008 showed their clinical benefits in patients with NYHA functional class III heart failure.^158^ The IN-TIME trial later tested a similar strategy using multiparametric monitoring devices (ICDs and resynchronization defibrillators). The parameters evaluated in the trial included events like ventricular and atrial tachyarrhythmia, low percentage of biventricular pacing, increased frequency of ventricular extrasystoles, decreased patient activity, and abnormal intracardiac electrogram. Abnormalities in these parameters triggered a structured phone contact. The group allocated to telemonitoring had a significant reduction in combined clinical outcomes and total mortality.^159^ Other similar studies have also shown a reduction in combined clinical outcomes, often related to a decreased need for face-to-face visits.^160^ Results from an unselected population cohort study also indicated benefits of remote monitoring with information from ICD/cardiac resynchronization therapy (CRT) on mortality.^161^ However, a meta-analysis^162^ of 11 randomized trials evaluating 5,703 patients showed no consistent results on clinical outcomes. The meta-analysis showed that device telemonitoring was associated with a reduction in the total number of planned, unplanned, and emergency room visits (RR 0.56, 95%CI 0.43-0.73, p < 0.001). However, rates of cardiac-related hospitalization (RR 0.96, 95%CI 0.82-1.12, p = 0.60), the composite endpoints of emergency visits, unplanned hospital visits, or hospitalizations (RR 0.99, 95%CI 0.68-1.43, p = 0.96), and total and cardiac mortality were also similar between groups.^162^

#### 2.7.8. Atrial Fibrillation

Patients with atrial fibrillation (AFib) comprise a special group, considering that, among other things, AFib has been accounted for approximately 60% of pacemaker and CRT/defibrillator (CRT-Ds) alerts and nearly 10% of all ICD alerts in a worldwide database.^163^ Remote monitoring has excellent sensitivity (95%) in detecting AFib, a feature that becomes even more important, considering that 90% of the detected episodes were asymptomatic.^164,165^ The potential benefits of remote monitoring include detection and early reaction (*e.g.*, drug therapy, device reprogramming, or electrical cardioversion) to prevent atrial remodeling and serious adverse events related to AFib. The IMPACT trial has shown that the detection of asymptomatic AFib via remote monitoring considerably shortened the time to anticoagulation initiation (3 days versus 54 days) but was not associated with reduced rates of stroke, systemic embolism, and bleeding.^166^

In the preclinical phase of an arrhythmia, telemedicine screening may detect asymptomatic AFib.^89^ In a pilot study, the daily transmission of electrocardiographic data by phone facilitated the diagnosis of asymptomatic paroxysmal AFib.^90^ In large cohorts, telecardiology services improved the management of patients with AFib and detected new cases of arrhythmia.^167^

Support by telemedicine can improve the diagnosis of silent AFib.^168^ Bilgi et al.^169^ demonstrated that home-based electrocardiographic assessment supported by telecardiology increased the sensitivity of the diagnosis of AFib in elderly individuals and was useful in identifying individuals with AFib and atypical symptoms at home.^169^ The electrocardiogram (ECG) was recorded and transmitted by a smartphone to a 24/7 telecardiology center and evaluated by a cardiologist. The telecardiology support increased two times (40 years), four times (60 years), and seven times (70 years) the rate of AFib diagnosed at home.

The SEARCH-AF study has shown that the use of an ECG lead (DI) on an iPhone ECG (iECG, AliveCor KardiaMobile) accurately diagnosed AFib, making this technology feasible for the screening of subclinical AFib in primary care and in the community.^170^ In the REHEARSE-AF study, a randomized trial of AFib screening involving 1,001 participants aged ≥ 65 years and with CHA^2^DS2-VASc ≥ 2,^171^ the participants were randomized to screening with AliveCor KardiaMobile (iECG) twice a week for 12 months (and additional ECG in case of symptoms) or usual routine. The use of iECG increased by almost four times the diagnosis of AFib (HR 3.9, p = 0.007).

Smartphones, apps, and cloud storage technology have the potential to change the practice of medicine and the way decisions are made. On smartphone platforms, medical or health care applications can analyze a range of vital signs through built-in sensors, interconnected devices, or peripherals.171 The transfer of ECG images by multimedia messaging can be a practical, low-cost procedure in telecardiology.^172^ These new technologies may increase the detection of arrhythmias, but the real value of these new methods has yet to be evaluated in rigorously conducted studies.

#### 2.7.9. Channelopathies

Inherited electrical syndromes are less frequent indications for ICD implantation. However, their management can be challenging because these devices are then implanted in patients who are often younger and less likely to comply with the required follow-up.^174^ Electrical abnormalities may occur in these diseases, predisposing the patient to unnecessary shocks and requiring careful programming.^175^ The pediatric population, which often has implanted epicardial electrodes, is specifically more vulnerable. In such patients, telemonitoring may be particularly useful for surveillance, early detection, and preventive programming.^176^ In the multicenter Brugada registry, the number of outpatient visits was significantly lower in a telemonitoring group compared with a control group (p < 0.001), and there was a trend suggesting that the number of inappropriate shocks was also reduced.^177^

#### 2.7.10. Tachycardia and Ventricular Fibrillation

Remote patient monitoring can be valuable for prompt assessment of the appropriateness of the detection and the efficacy of the administered therapy. If shock is appropriate, and clinical condition is stable, the physician can reassure the patient without requiring a hospital visit. In a multicenter pilot study, 81% of the episodes of ventricular tachyarrhythmia were analyzed remotely, and in 85% of the cases, no further action was required.^178^ The TRUST study demonstrated that remote monitoring allows early detection of ventricular tachyarrhythmias compared with standard follow-up (1 day versus 36 days for ventricular fibrillation and 1 day versus 28 days for ventricular tachycardia, p < 0.001).^179^ Other potential benefits of remote monitoring include the prevention of inadequate shocks and appropriate but unnecessary shocks. Inadequate detection due to supraventricular tachyarrhythmias (or T-wave oversensing) may lead to the patient receiving a notification for in-hospital reprogramming or other interventions.^180^ Proper delivery of ICD shock for slow, stable ventricular tachycardia may lead to device reprogramming with broader use of painless antitachycardia therapies. Recurrent and self-limited asymptomatic rapid ventricular tachycardia occurring in the ventricular fibrillation window (triggering alerts in some systems, regardless of the administered therapy), can be detected early and, with appropriate intervention, be programmed to prevent electrical storms. In addition, timely treatment of tachycardias may prevent early battery depletion caused by recurrent loads and shock administration.^176,181^

#### 2.7.11. Congenital Heart Disease

Tele-echocardiography can establish an early diagnosis of congenital heart diseases to guide therapeutic management.^182^ A North American multicenter study in 338 paired infants (with and without access to telemedicine) with or without minor heart disease showed a statistically significant reduction in the percentage of infants transferred to a tertiary hospital (10% versus 5%), as well as in total hospital stay and intensive care unit (ICU) stay.^183^ Telemedicine increases the ability of pediatric cardiologists to provide higher quality care to a greater number of patients, although high-quality studies evaluating the impact of this intervention are still limited.

### 2.8. Cardiovascular Teletomography and Teleresonance

Despite the apparent similarity between teleimaging and local diagnostic services, divergences between both occur in one fifth of the cases. Specifically, divergences with clinical impact occur in up to 1 to 3% of the cases. We can hypothesize that the reasons for these divergences may be inadequate imaging quality, unavailability of patients’ clinical data (like current and past history and physical examination), limited access to patients’ laboratory tests and other imaging evaluations, fatigue, and simple interobserver variation.

The workflow of imaging diagnosis in local hospitals and in telediagnosis may be difficult to distinguish. Generated images are stored in imaging systems (picture archiving and communication system - PACS, for example) and then analyzed by the radiology department (and other specialties working with imaging tests, like cardiology and obstetrics). In teleimaging, the image is transmitted to an external center and analyzed the same way that it would be done locally. Further studies are needed to compare the diagnostic performance of teleimaging versus local imaging. Both may even be assumed to have the same performance, but evidence is critical to confirm our assumptions and can determine not only whether teleimaging can be performed, but also the optimal conditions to be carried out safely and cost-effectively without harm to the patient.

#### 2.8.1. DICOM Standard

A new group of services developed in previous years was introduced in 1993, the Digital Imaging and Communications in Medicine (DICOM) standard. The objective was to standardize data and information obtained by imaging methods, normalizing the rules for transmission and storage of medical information. This group of services uses a digital format that associates images with metadata-type information with the ability to optimize search and exchange of information and the use of images by specific software programs. DICOM specifications are updated from time to time without losing sight of previously established functionalities.^184,185^

#### 2.8.2. MRI, CT, and Telemedicine

Contrast-enhanced magnetic resonance imaging (MRI) and computed tomography (CT) scanning bear additional complexities, requiring specific care at centers performing these tests. These range from the scheduling of the tests - in which there is a need to define its precise indication, *e.g.*, pharmacological stress test (dipyridamole, adenosine, dobutamine), evaluation of myocardial ischemia, viability, valvular heart disease, specific cardiomyopathies, among others - to the need of on-site physicians due to frequent use of contrast and medications, nursing technicians to obtain an adequate venous access, and biomedical doctors and technologists with specific training and experience in the acquisition and postprocessing of MRI and CT scan images using software dedicated for these analyses.

The images should be read by experienced specialized physicians with specific training in that particular area of diagnostic imaging. Such training usually requires 2 years and is not widely available nationwide, limiting the number of trained specialists for appropriate MRI and CT scanning.

The complexity of performing MRI and CT scans with remote guidance and reading, together with the need for specialists to analyze the images and the possibility of the test being performed at any given time, depending on the clinical indication, makes telemedicine increasingly important for this activity.

#### 2.8.3. The Federal Council of Medicine and Tele-CT/Tele-MRI

In 2014, the CFM updated the rules for tele-CT/tele-MRI practice in Brazil.^186^ These rules are valid for the transmission of patients’ images between different locations to produce a medical report, a second expert opinion, or a clinical imaging review. The rules related to the topic of this document are listed below:

- Clinical data - The transmission of tests by tele-CT/tele-MRI should be accompanied by necessary clinical data of the patient, collected by the requesting physician, for the preparation of the report.
- Patient authorization - The patient must authorize on an informed consent form the transmission of images and data.
- Local and remote specialist - The responsibility for the remote transmission of tests and reports must be assigned to a specialist in MRI and CT scanning.
- Limits for remote practice - In the case of noncontrast imaging (*e.g.*, calcium score - CS), and in the absence of a specialist physician at the health care facility, the attending physician may ask the specialist for appropriate remote diagnostic support.
- Specialist required - A specialist physician must be present in centers where contrast imaging tests - including MRI and CT scans - are performed.
- Shared responsibility - The professional responsibility for the care lies with the specialist physician caring for the patient undergoing the test. The specialist issuing the remote report shares this responsibility.
- Headquarters in Brazilian territory - Legal entities providing services in tele-CT/tele-MRI must be headquartered in Brazilian territory and be registered with the CRM of their jurisdiction. If the provider is an individual, he or she must be a physician trained in MRI and CT scanning.
- Operating standards - This is beyond the scope of this document, but specific information can be found in another document on operating standards and minimum requirements for the transmission and handling of remote imaging diagnostic reports.
- Image compression and transmission - Communication protocols, file formats, and compression algorithms should comply with current DICOM and HL7 standards. The specialist physician is responsible for evaluating the compression ratio.
- Image visualization and processing - The specialist physician is responsible for ensuring the technical characteristics of remote workstations, monitors, and ergonomic conditions in order to avoid compromising the diagnosis.
- Safety and privacy - Computerized systems used for the transmission and handling of clinical data and diagnostic imaging reports and for sharing of image and information must comply with CFM regulations. Specifically, tele-CT/tele-MRI, systems must meet the mandatory requirements of the “Level of Safety Assurance 2 (*Nível de Ga*rantia de Segurança 2, NGS2)” established in the current Certification Manual for Electronic Health Registration Systems issued by the CFM and the Brazilian Society of Health Informatics (*Sociedade Brasileira de Informática em Saúde*, SBIS).

#### 2.8.4. Imaging Transmission

Imaging transmission must comply with CFM standards regarding quality and security. However, MRI and CT images are generally larger, requiring an infrastructure with adequate data bandwidth for transmission. Importantly, the specialist physician responsible for the report must download the images. Thus, the choice of important sequences after image acquisition and the method of compression are fundamental for a smooth flow.

#### 2.8.5. Postprocessing Software and Workstations

Ranging from ECG images to three-dimensional coronary angiotomography (TCA) images and the wide variety of MRI sequences, several imaging parameters must be evaluated, most requiring specific software.

*Assessment of CS -* CS is assessed with software programs installed in workstations, which are usually purchased with the CT equipment, or with other independent programs or plug-ins. The report usually informs the amount of calcification in each artery along with the total CS value and percentile for the patient’s gender and age, based on several population studies, of which the most used and recommended is the MESA (MultiEthnic Study of Atherosclerosis) study.^187,188^ Other data such as arterial age and the patient’s overall cardiovascular risk variation based on the ECG can also be described.

*Evaluation of TCA -* After the appropriate acquisition phase, aiming at the best temporal and spatial resolution of the coronary arteries, and editing of the ECG, which is usually done directly in the CT equipment, other applications are helpful in establishing a diagnosis.

Until the requesting physician is able to obtain a clear view, the interpretation of the findings may be helped by software programs that extend the coronary arteries in a single plane (curved planar reconstruction - CPR), visualization of three-dimensional rendered images (using a 3D-volume-rendering technique), visualization of bidimensional images with multiple oblique planes (multiplanar reconstruction - MPR), and the characterization of coronary plaques, as well as the objective measurement of stenoses.^189^

*Evaluation with MRI -* As one of the most versatile imaging methods available, MRI is able to produce images of almost any anatomical plane and provide a wide range of pulse sequences to generate images with specific characteristics, allowing assessments that range from the evaluation of ventricular function to myocardial tissue characterization.^190^ Several software applications are available for this purpose, including applications for:

- assessment of volume, ventricular mass, and right and left cardiac function;
- analysis of intracardiac flow to measure QP-QS, shunts, and valvular dysfunction;
- magnetic resonance angiography postprocessing with measurement of vascular diameters;
- tissue characterization to quantify perfusion, necrotic/fibrotic mass, iron deposition by T2* evaluation, and parametric maps of T1, T2, and T2* values.

The strategy of using software programs in MRI and CT imaging is strongly recommended and can improve the time required to read the images, the accuracy of the reading, and the clarity of the report of the findings.

#### 2.8.6. Database, Communication, and Image Archiving

The integration between radiological information system (RIS) and PACS enables the assignment of a unique registration for each patient. This optimizes the information by combining images with clinical data and making the process faster and more secure. This format has been increasingly used in health care centers and often enables remote access, facilitating the use of tele-CT/tele-MRI and improving administrative procedures and communication.

Several solutions are available in this regard, including cloud-based web solutions. Remote access to images and the ability to distribute reports via a standard universal system are helpful for the workflow.

A report is nothing more than a type of communication with the main objective of transmitting the assessment of images analyzed by an expert to another physician who needs such information to make decisions. The more complete and clear the transmitted information is, the more important the requested test becomes. The development of structured reports linking written information to tables, figures, and photos to make the information as clear and accurate as possible is an ongoing trend.

As described earlier, reports may be made available through advanced systems like RIS, but other forms of transmission, including instant messaging applications like WhatsApp, may be used. According to the CFM,^191^ WhatsApp and similar platforms can be used for communication between physicians and patients, as well as privately between physicians for transmission of data or questions, or in closed group chats between specialists or clinical staff of an institution or chair, provided that all information transmitted is absolutely confidential, remain within the group, and is not circulated to recreational groups, even if these are composed only of physicians.

#### 2.8.7. Clinical Indications for MRI and CT

Interestingly, no studies in the literature have assessed the clinical impact of the application of tele-MRI or tele-CT. Thus, clinical recommendations in this guideline are based on level C evidence, including expert consensuses, and in the absence of studies evaluating cost-effective outcomes. Aware of this limitation, we cite at the end of this document the main indications for the application of tele-CT/tele-MRI in this subarea of cardiovascular imaging.^192^

The use of MRI and CT imaging has been increasing, and characteristics of these imaging methods make their use very interesting in telemedicine, particularly in countries with continental proportions like Brazil and in those with a limited number of available MRI/CT specialists. The possibility of having a specialist potentially accessible at any moment can be helpful in patient management and in lowering health care costs by optimizing the time of available specialists and expediting reports of hospitalized patients, which can shorten their hospital stay, and in other applications related to this medical progress.

## 3. Telerobotics Applied to Cardiology

### 3.1. Robotic Telesurgery

The concept of telesurgery was introduced in the early 1970s by NASA.^193^ The objective of the original project was to provide medical care to astronauts during remote missions.

Robotic telesurgery devices are applications in which the surgeon controls remotely a robot that executes the surgical procedure. The da Vinci® system (da Vinci® surgical system; Intuitive Surgical, Sunnyvale, CA, USA), the most widely used robotics platform today, follows this approach. The surgeon works on a console separated from the surgical field, and the movement of his or her hands is perceived and transmitted to the instruments close to the patient. This technique yields great ergonomic benefit to the surgeon, incorporates functions like hand tremor cancellation, and broadens (in three dimensions) the view of the field that the surgeon is interested in. However, these platforms lack much automation and require continuous involvement of a human operator (surgeon) for regulatory reasons.

ARTEMIS was the first surgical robot used for cardiac procedures.^194^Designed as a telesurgery and telepresence system for cardiac procedures, it was used for training and planning and to perform minimally invasive procedures.

Currently, robotic cardiac surgery has been used primarily for mitral valve repair and myocardial revascularization, following approval by the Food and Drug Administration (FDA) in 2002 and 2000, respectively. Newer techniques assist in cardiac manipulation procedures by compensating heart movements. However, large-scale implementation of this technique is hampered by its high cost^195^ and the absence of randomized studies demonstrating its superiority over traditional minimally invasive techniques, with or without hybrid procedures.^196^

The first robotic mitral valve heart surgery was performed in 1998 by Carpentier, in France, and Mohr, in Germany. That same year, Carpentier conducted the first coronary artery bypass surgery in Paris, while Reichenspurner performed revascularization surgery with the voice-controlled ZEUS Robotic Surgical System (Computer Motion, Goleta, CA) in Munich.^196,197^ Since then, this technique has become popular as it is associated with less surgical aggression, reduced cardiopulmonary bypass and aortic clamping duration, and shorter hospital stay compared with the conventional open technique.^197^

The da Vinci® robotic system has allowed improved visualization of the surgical field with three-dimensional capture and ten-fold magnification, resulting in greater precision in the surgical procedure and smaller incisions following a learning curve.^198,199^ It has also reduced the rates of all complications (particularly infection), blood transfusion, and time to return to work activities, with an impact on the total costs of the procedure. This has been observed especially among patients at high risk (like elderly individuals) and those with ejection fraction lower than 20%, diabetes of difficult control, and severe chronic obstructive pulmonary disease.^200^ This robotics system has also benefited hybrid surgeries, angioplasty, and minimally invasive procedures in patients with multilateral obstructive coronary artery disease.^201^

Both computer-integrated surgery and telemedicine are becoming popular in the developed world. Advancements in robotic technology and information technology, like the Internet of Things, allow robotic arms to be controlled remotely, enabling robotic telesurgery. With telesurgery, surgeons can perform surgical procedures in remote locations, away from the patients, improving the access to medical treatment and, potentially, the quality of the treatment.

As with other telemedicine applications, telesurgery can broaden the access to interventions in remote areas or centers where specialists in particular types of surgery are not present, for example. The importance of telemedicine, telesurgery, and remote surgery is not restricted to their ability to perform medical procedures in areas where these procedures are not available and can be extended to telementoring, which involves the training of medical professionals to perform these procedures.^202^ In this area, telesurgery could also benefit patients requiring infrequent, highly complex interventions, in which the medical-surgical expertise is not widely available. The acquisition of new technology expertise by specialists has been accompanied by mentoring programs (proctors). In robotic surgery, telemedicine can provide training with remotely connected proctors (telementoring), expanding the access to innovative technologies.^203,204^

Outcomes of robotic surgeries still lack long-term analyses of hard outcomes like all-cause mortality, cardiovascular death, fatal AMI, stroke, need for repeat revascularization, and vascular graft patency. As in traditional surgeries, patient selection is essential, and the goal should be complete revascularization, bearing in mind that CO_2_ insufflation decreases venous return by increasing intrathoracic pressure and may compromise hemodynamic parameters in patients with left ventricular dysfunction and in those with chronic obstructive disease, who would benefit more from minimally invasive surgery. Case series have been reported totaling about 110 patients and showing 90% of surgical success within 30 days without open surgery and a maximum follow-up of 5 years.^205-207^

The largest experience in this area has been published by Cavallaro et al.,^208^ who reported rates of morbidity and mortality with robotic myocardial revascularization surgery in 2,582 patients between 2008 and 2010. The authors reported lower rates of postoperative complications in selected patients but concluded that these benefits decreased in patients requiring multiple bypass grafts.^208^

Approximately 1,700 robotic heart surgeries are performed annually in the US, including 35.5% for mitral valve repairs.^209^ In 2011, the FDA introduced a post-marketing surveillance plan known as the Medical Device Epidemiology Network initiative, leveraging AI to real-world data analysis, including international registries and electronic medical record data, to bridge the gap in evidence gathering concurrent with the implementation of technological innovation without compromising patient safety.^210^

In Brazil, none of the robotic surgical techniques have been included in the public policy list or in the List of Procedures and Events in Health of the National Health Agency.^210^ In this sense, the CFM, through Resolution 2.227/2018, which was later repealed, had regulated robotic telesurgery, precisely anticipating the expansion of the benefits of the technique and facilitating the follow-up of the learning curve by proctors in remote locations. Thus, robotic surgery in Brazil can be remotely assisted by a specialist at a large center in another country. Although the CFM allowed the use of robotic telesurgery in Brazil, it restricted the method to professionals qualified to practice medicine in the country.^39^ Of note, mentoring/proctoring programs in Brazil have not been widely regulated, except in the State of Paraíba, where the CRM, through Resolution CRM-PB 182/2018, defined rules that regulate and legally secure the practice to the medical act.^39^

In addition to the information presented above, synchronization between the vast potential of these technologies and the existing ethical and legal apparatus is also lacking. Unlike a comprehensive national policy, there is a general scenario of fragmentation characterized by different norms and standards issued by different agencies and with a different focus.^7^ Even if a single instrument could hardly achieve these objectives, fragmentation would be yet another obstacle to overcome to reach the potential that telemedicine and telesurgery have in our country.

Among other barriers to their practical use are the scarcity of resources and technical knowledge, as well as infrastructure issues. Brazil is a country of unequal regional distribution in terms of broadband availability.^7^ This means that the infrastructure of the broadband data network is one of the most limiting factors for the expansion of telemedicine in general and telesurgery in particular, especially in rural areas of the country.

### 3.2. Robotic Angioplasty

PCI can be considered a highly predictable, safe, and minimally invasive therapy. However, this manual and operator-dependent procedure must be executed in person, demanding physical action by the physician. Full proficiency can only be achieved in high-volume environments with scenarios involving highly complex and technological interventions. Coronary angioplasty also exposes both professional and patient to ionizing radiation. As a result, the potential risks are high for occupational health damage arising not only from the radiation but also from the need of individual protection^211-213^e.g., a 7-kg lead apron).

Robot-assisted coronary interventions have recently been developed as an alternative to reduce the reliance on manual operation,^214-224^ potentially reducing damage from radiation exposure.^225^ Clinical studies have demonstrated the safety and efficacy of the robotic system, which has already been approved for routine application in the US and the European Union. Even though the current set of scientific evidence is encouraging, it is also recent and limited to the number of patients treated, hindering further consideration of possible risks and benefits of the technique, especially when it comes to particularities of its application in subgroups of clinical interest.

## 4. Telemedicine for Provision of Services and in Supplementary Health

### 4.1. Provision of Services

Over 60% of all health care facilities and between 40-50% of all US hospitals are currently estimated to use some form of digital data transmission.^226^ In 2016, a US health care facility reported that communication of digital health data (e-mail, phone, and video) exceeded the number of in-person consultations.^227^

However, it is important to note that despite new modes and means of communication transmission between physicians and patients, ethical and legal responsibilities remain the same as those governing the traditional physician-patient relationship.^228^ Cardiologists must inform their patients about telemedicine services and their limitations, the possibility of late follow-up, by encouraging regular reporting to their attending physicians, and how they can receive electronic health-promoting information.

Reimbursement is a key determinant of the success of clinical interventions. The movement toward value-based reimbursement rather than payment for service, which provides incentives for care in lower-cost settings, along with the identification and interaction with high-risk individuals before disease onset, and the efficient use of integrated care teams, provide incentives for telemedicine expansion. Understanding the effect of reimbursement within the context of alternative payment models is a priority.

While the path of value-based reimbursement is uncertain, the efficiency of care will inevitably be a priority in any scenario. Ensuring that these technologies are used for patients who meet the appropriate clinical requirements is also an important related topic. In the US, reimbursement for medical services by telemedicine has gradually expanded from coverage of services provided in rural settings to a broader program (Medicare Access and CHIP Reauthorization Act).^229^ Training and development projects have been created in Brazil, along with a continuing medical education with special attention to the SAMU/UPA care model, developed by the Ministry of Health and private hospitals.^230^

Regarding the payment for telemedicine and telehealth services in Brazil, as already mentioned, it should be considered that the main source of funds has been the public sector:^231-233^

- public communications from national (such as the National Council for Scientific and Technological Development - CNPq - and the Financier of Studies and Projects - FINEP) and regional research and innovation funding agencies (state research supporting foundations);
- agreements or direct transfer of funds to universities and health departments, within the scope of the program *Telessaúde Brasil*, then *Telessaúde Brasil Redes*;
- service providing agreements between public administrators and telehealth centers at university centers;^232^
- projects within hospitals receiving tax waiving by the PRO-ADI SUS program from the Ministry of Health.^215^

Many of these investments occurred at an early stage of technological development in the country, and only some of the fostered nuclei became active, sustainable services.^232^ There is a clear need for inclusion of telehealth procedures in the list of procedures paid by the SUS in order to regulate and encourage their routine use in the health system.

Supplementary health, in turn, lacks formal mechanisms of payment and reimbursement for telehealth activities, so telemedicine actions in this sector have been focused on optimizing care and reducing costs, and are often associated with specific conditions like stroke.^231^

Of note, the Ministry of Health Secretariat of Science, Technology, and Strategic Inputs, through Ordinance 26, of August 2, 2017, made public the decision to incorporate remote monitoring technology for the evaluation of patients with cardiac implantable electronic devices (CIEDs) within the scope of the SUS. This is an unprecedented incorporation of remote monitoring technology as a result of industry demand. The requester assessed the budgetary impact of the technology over 5 years, considering only direct expenses with the purchase of the remote monitoring device and the provision of conventional monitoring in a base case. A second scenario analyzed a model of dynamic transition state to account for opportunity costs of both technologies, exploring the advantages and disadvantages of each strategy. With the decision, the technology should have been included in the SUS within 180 days from the publication of the incorporation order by the Secretary of Science, Technology, and Strategic Inputs, but to date, the Clinical Protocol and Therapeutic Guidelines making this technology available in the SUS have still not been published.^234^

Unfortunately, there are still several gaps in the process of recognizing medical services in telemedicine for the purpose of reimbursement. The example above is the only one deliberated for public health. The process of coding hierarchical classifications or other payment modalities for the various telemedicine services has not yet been properly structured.

A more complete and structured set of codes would also provide more accurate data to address the scarcity of systematic economic evaluation of the benefits of telemedicine in both pay-per-service and value-based models.^235^ Bridging this gap is essential to guiding public and private health care providers and technology buyers and investors on decisions about investment returns in this field.

### 4.2. Telemedicine in Supplementary Health

In 1988, Brazil opted for the establishment of a universal health care system free and fully accessible, pursuant to art. 196 of the Federal Constitution. However, health care is available to private initiative, as established in the Magna Carta (Paragraph 1 of Art. 199) pertaining to the scope of participation of the private initiative: “Private institutions may participate in a complementary way in the SUS, according to its guidelines, by means of a contract of public law or agreement, with preference given to philanthropic and nonprofit entities.”^236^

Supplementary health assists over 47 million beneficiaries in Brazil and is governed by its own legislation, regulated by the National Health Agency. This is a health care system with its own characteristics and regulatory framework guided by specific legislation (Law No. 9.656/1998). The benefits gained from the remarkable development of ICT also apply to supplementary health. However, it should be noted that, due to particularities of laws governing the sector, health care plan operators are required to offer a myriad of procedures included in the List of Procedures and Events in Health of the National Health Agency.^237^

Also worthy of note is the fact that when one buys a health care plan, priority is given to access to physicians and therapies that supplement the list of policies offered by the SUS. Most of the supplementary health beneficiaries live in large centers, with over 35 million beneficiaries in the Southeast (28,650,281) and South (6,912,748) regions and more than 18 million in capital cities. An analysis of the intersection of demographic data of supplementary health beneficiaries and Brazilian physicians, including cardiologists, shows that the proportion of physicians per inhabitant (beneficiaries of supplementary health) in these regions is different from the one observed in public health, where truly remote areas receive no coverage. It is imperative to consider that the in-person relationship between physician and patient is the rule in supplementary health, which does not hinder the possibility of making telemedicine resources available.^238^

Accordingly, the various services provided by telemedicine are fully applicable to supplementary health. However, the provision of face-to-face consultations with experts rather than their “tele” versions is a legal demand. ICT resources should not be offered to replace face-to-face interaction but should be an option to improve care also in the context of supplementary health.

Increased efficiency in health care requires quality improvements and costs savings.^239^ The integration of telemedicine into outpatient clinics and hospitals, including supplementary health, can help achieve both goals.^240^

Medical care is, without question, one of the most (if not the most) complex sectors in the economy. Considerable financial investment and years of persistence are required to build an effective telemedicine system. The widespread adoption of this type of practice also requires behavioral adaptations by many physicians, organizations, and patients.^241^ The industry in this area still requires better regulation.

Telemedicine can be an affordable alternative to meet the health needs of vulnerable populations with multiple comorbidities requiring frequent care.^242^ Improving patient engagement, telemedicine can provide an effective platform for patients to engage in their own decisions.^243^ For example, the US Veterans Health Administration introduced a national telemedicine program named Care Coordination/Home Telehealth. This model allows patients to manage their own conditions,^244^ and some important studies have reported that the shared-decision model has reduced hospitalization rates.^245^

Unequal access to health, even in supplementary health, is persistent in Brazil and requires major investments to improve the organization of health care systems. Even when health care services and evidence-based guidelines are available for common and relevant conditions like hypertension and diabetes, the implementation gap is vast, and best practices are not absorbed by health care professionals, or recommended measures are not followed by patients and their relatives. The science of implementation has proved to be as important as data analysis in recent decades for the recognition of bottlenecks preventing full use of preventive and therapeutic measures to ensure benefits for patients who can live longer and better by taking advantage of all available knowledge,^246^ reducing costs and increasing the efficiency of private health care systems.

Direct physician-patient teleconsultation, not yet regulated in Brazil, is the most frequently used model. A US report showed that telecardiology was useful for evaluating both new and recent postoperative referrals, with the potential benefit of knowledge transfer to local primary care. In Canada, teleconsultation has been useful for the evaluation of new patients with syncope and supraventricular tachycardia.^247^ In the United Kingdom, a wide range of inpatient and outpatient telecardiology services is available at district hospitals using various technologies.^248^ This approach has improved access, was cost neutral, and increased patient satisfaction. The authors emphasized that it complemented but did not replace regular consultations.

The demand for home care using web-based applications directed to consumers, including tablet and smartphone applications, is growing exponentially.^249^ Many health care providers worldwide are adopting this technology as a way to provide low-cost care for common problems that could result in a visit to the emergency department.^250^ Most of these applications rely solely on video and audio connections with additional software for scheduling, billing, sharing of still images, and documentation. Also, some peripherals, such as smartphone-compatible heart rate sensing devices,^251^ are available for purchase at decreasing prices.

Limited financial availability for the acquisition and maintenance of telemedicine equipment and infrastructure remains a barrier to the widespread deployment of telemedicine.^252^ This is particularly true for many health care providers or small systems that may lack the required resources and often have conflicting demands for available funds. Other costs associated with telemedicine programs include salary, administrative support and supplies, software and application development and upgrade costs, training programs, and initiatives to promote the program to patients.^253^ With increased mobile connectivity, smartphones, and video compression, the costs to implement simple telemedicine interactions have decreased.

Currently, none of the procedures used in telemedicine are included in the Supplementary Health List of Procedures and Events in Health, *i.e.*, a regulatory vacuum current places telemedicine in a field of conjecture and frustrated expectations dissociated from the interest of the regulated sector. There is also no concrete scientific evidence available to support the formal use of this technology.^38^

Telemedicine is a disruptive innovation with the potential to change health, and its influence is likely to increase rapidly. Guided by what is best for patients, telemedicine, if properly applied, will help usher in a new age for health care that will be built by patients and physicians, identifying new ways of care, increasing quality and rationalizing costs also in supplementary care.^240^

## 5. Economic Evaluation and Budgetary Impact of the Incorporation of Telemedicine in Cardiology in Brazil

### 5.1. Concepts of Economic Evaluation in Telemedicine

The implementation of telemedicine services seeks to provide accessible and quality health care at a low cost.^254^ Since the 1990s, as technological communication capabilities advanced, telemedicine services became more prevalent.^255^

The emergence of new telemedicine-related capabilities and their integration into care systems offers opportunities to enhance value-based clinical care, promote health, and prevent diseases.^256^

The values associated with the adoption of telemedicine services include the collaboration with the agile accessibility of patients to highly complex centers,^257^ along with a reduction in mortality^258^ and frequency of hospitalizations.^259^

In cardiology, recent publications portray telemedicine and teleconsultation services as effective in the management of patients with chronic heart failure,^260,261^ preparation of ECG reports, guidance of patients through mobile applications,^262^ and cardiac rehabilitation,^263^ among others.

From reported experiences, telemedicine is universally seen as offering interesting benefits for improving accessibility and quality of health.^264^ However, scientific and financial investments required to introduce these technologies into the health care system are high,^265^ which potentiates the importance of accurate studies on economic analysis to guide decisions upon implementation of telemedicine services.^265,266^

Economic analysis is one of the pillars of health care assessment that aims to support and guide decision making. For a new technology to be applied to a process, it must replace existing alternatives with equal or better results. That is, it must be effective. In addition, the results thus obtained should be less expensive than the alternatives or present reasonable values related to benefits, i.e., it must be cost effective.

However, the economic evaluation of telemedicine strategies has some specificities: constant change of technology, lack of an adequate study design to manage undersized samples, inadequacy of conventional economic assessment techniques, and health outcome assessment problems not directly related to health.^267^ As a consequence, different types of cost analysis have gained an important role in the evaluation of telemedicine services.

### 5.2. Applied Economic Methods

Different approaches have been used to assess the economic impact of telemedicine services with varying levels of acceptability. Basically, five methods are available for calculation of cost effectiveness between conventional and telemedicine interventions: cost-minimization analysis, cost-benefit analysis, cost-effectiveness analysis, and cost-utility analysis. Additionally, return on investment has been used in telemedicine projects.^268^

Cost-minimization analyses assume that both alternatives (conventional and telemedicine) are equally effective in health outcomes but differ in cost. A cost-minimization analysis considers changes but keeps health outcomes unaltered. For example, telecardiology for ECG reports assumes the same result but with different costs. Consequently, managers have to consider only differences in costs when deciding which alternative is less expensive.^269^

Cost-benefit analysis recognizes that different projects are equally effective, but results and costs change. Changes in health costs and outcomes are considered simultaneously and attribute a monetary or numerical value not only to costs but also to health outcomes in order to express the nondimensional cost/benefit ratio. However, the idea of attributing monetary values to health outcomes, such as years of life, is not always acceptable to health decision makers.

Cost-effectiveness analysis appears as a solution when costs and results are considered simultaneously, without attributing monetary values to results. Each outcome is defined according to its specific unit so that the final indices demonstrate a relationship between economic and health outcomes. Therefore, the final decision depends on the relationship that the decision maker considers best. This ratio (incremental cost-effectiveness ratio, ICER) represents the cost for each additional result unit.^252^

Cost-utility analyses consider individuals’ quality of life and the time of life that they will obtain as a result of an intervention. This is a particular case of cost effectiveness in which results are measured in terms of full-life lived years, usually expressed in quality-adjusted life-years (QALYs) or disability-adjusted life-years (DALY). The WHO recommends a value for DALY equivalent to three times the gross domestic product per capita.

Return on investment is a nondimensional relationship between the monetary value invested and the monetary gain resulting from these investments, and measures the efficiency of the investment.^270^

### 5.3. Literature Review

A systematic literature review of cost-benefit review studies on the adoption of telemedicine services was conducted by the Federal University of Minas Gerais (UFMG) in 2016, gathering publications from 2000 to 2016.^105^ Considering the keywords employed in the study, a search was performed on the PubMed database with the inclusion criterion being only studies related to cardiology. Other reviews manually searched in databases and/or journals of the area were also added. Thus, the search included literature review articles on the assessment of the incorporation of telemedicine in cardiology from 2000 to April 2019.

Variables related to the economic analysis method, purpose of telemedicine in cardiology, clinical effectiveness, and cost reduction were extracted from the studies. For effectiveness, the impact on the reduction of mortality and hospitalizations, improvement of medication management, and anticipation of diagnosis were evaluated.

**Figure 5.1:**
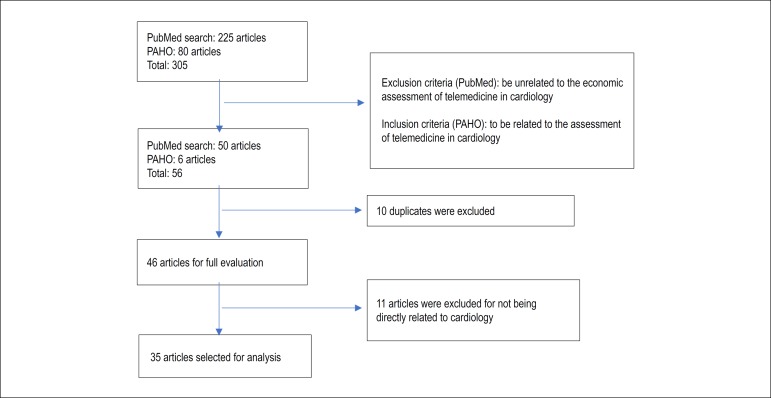
Flowchart of selection of articles on economic analysis of Telemedicine applied to cardiology.

From the search and application of exclusion criteria, 35 articles were fully analyzed by two researchers (A.E. and B.Z.) for the collection of variables. Figure 5.1 presents the flowchart of the selection of the articles.

The number of studies focusing on telemedicine, especially the monitoring of chronic diseases, has increased over the last decade. Of the 35 studies identified, 19 were published after 2015. Table 5.1 summarizes the characteristics of the articles.

**Table 5.1:** Studies on economic evaluations of telemedicine in cardiology

**#ID**	**Authors**	**Year**	**Number of papers assessed**	**Category of telemedicine services**	**Service focus**	**Reduction in mortality**	**Reduction in hospitalizations**	**Reduction in hospital stay**
1	López-Villegas A, Catalán-Matamoros D, Martín-Saborido C, Villegas-Tripiana I, Robles-Musso E.^284^	2016	7	Telemonitoring (mHealth)	Heart failure	N/A	N/A	Yes
2	Cajita MI, Gleason KT, Han HR.^271^	2016	10	Telemonitoring (mHealth)	Heart failure	N/A	N/A	N/A
3	Louis AA Turner T Gretton M Baksh A Cleland JG.^270^	2003	24	Telemonitoring (mHealth)	Heart failure	Yes	Yes	Yes
4	Kassavou A, Sutton S.^254^	2018	17	Mobile and automated communication	Guidance on medication use	N/A	N/A	N/A
5	Lin MH, Yuan WL, Huang TC, Zhang HF, Mai JT, Wang JF.^264^	2017	39	Telemonitoring (mHealth)	Chronic heart failure	Yes	Yes	Yes
6	Kotb A Cameron C Hsieh S Wells G.^285^	2015	30	Comparison between telephone teleconsultation services	Heart failure	Yes	Yes	Yes
7	Yun JE, Park JE, Park HY, Lee HY, Park DA.^286^	2018	37	Telemonitoring (mHealth)	Heart failure	Yes	Yes	N/A
8	Seto E.^269^	2008	9	Telemonitoring (mHealth)	Heart failure	N/A	N/A	N/A
9	Grustam AS, Severens JL, van Nijnatten J, Koymans R, Vrijhoef HJ.^260^	2014	22	Telemedicine	Chronic heart failure	Yes	Yes	N/A
10	Lee M, Wang M, Liu J, Holbrook A.^287^	2018	7	Teleconsulting	Guidance on medication use	Yes	N/A	N/A
11	Klersy C, De Silvestri A, Gabutti G, Raisaro A, Curti M, Regoli F, et al.^288^	2011	17	Telemonitoring (mHealth)	Heart failure	N/A	Sim	Não
12	Conway A Inglis SC Clark RA.;^289^	2014	25	Telemonitoring (mHealth)	Hypertension	Yes	Yes	Yes
13	Duan Y, Xie Z, Dong F, Wu Z, Lin Z, Sun N, Xu J.^290^	2017	46	Telemonitoring (mHealth)	Hypertension	N/A	Yes	N/A
14	Kitsiou S Paré G Jaana M.;^291^	2015	15	Telemonitoring (mHealth)	Chronic heart failure	Yes	N/A	N/A
**#ID**	**Reduction in hospital stay**	**Early diagnosis**	**Increased adherence to therapy**	**Not conclusive**	**Cost reduction**	**Most frequent economic analysis method among the articles**		
1	Yes	Yes	N/A	N/A	Yes	Cost-effectiveness		
2	N/A	N/A	N/A	Yes	N/A	N/A		
3	Yes	Yes	N/A	N/A	Yes	Cost-effectiveness		
4	N/A	N/A	Yes	N/A	N/A	N/A		
5	Yes	N/A	N/A	N/A	N/A	N/A		
6	Yes	N/A	N/A	N/A	N/A	N/A		
7	N/A	N/A	Yes	N/A	N/A	N/A		
8	N/A	N/A	N/A	N/A	Yes	Cost-minimization		
9	N/A	N/A	N/A	N/A	Yes	Cost-effectiveness		
10	N/A	Yes	N/A	N/A	N/A	N/A		
11	No	N/A	N/A	N/A	Yes	Cost-effectiveness		
12	Yes	N/A	N/A	N/A	N/A	N/A		
13	N/A	Yes	N/A	N/A	N/A	N/A		
14	N/A	N/A	N/A	N/A	Yes	N/A		
15	Polisena J Tran K Cimon K Hutton B McGill S Palmer K^292^	2010	21	Telemonitoring (mHealth)	Congestive heart failure	Yes	Yes	Yes
16	Pandor A Thokala P Gomersall T Baalbaki H Stevens JW Wang J.^266^	2013	2	Telemonitoring (mHealth)	Heart failure	Yes	Yes	N/A
17	Inglis SC Conway A Clark RA.; Cleland JG^285^	2015	27	Teleconsulting	Heart failure	Yes	Yes	N/A
18	Liu S, Feng W, Chhatbar PY, Liu Y, Ji X, Ovbiagele B.^286^	2015	13	Telemonitoring (mHealth)	Risk of stroke	Yes	N/A	N/A
19	Knox L, Rahman RJ, Beedie C.^287^	2017	26	Telemonitoring (mHealth)	Chronic heart failure	Yes	Yes	Yes
20	Pandor A Gomersall T Stevens JW Wang J Al-Mohammad A Bakhai A.^288^	2013	21	Telemonitoring (mHealth)	Heart failure	N/A	Yes	N/A
21	Hamilton SJ, Mills B, Birch EM, Thompson SC.^255^	2018	9	Telemonitoring (mHealth)	Cardiac rehabilitation and heart failure	N/A	N/A	N/A
22	Inglis SC Clark RA Dierckx R Prieto-Merino D Cleland JG.^289^	2015	25	Telemonitoring (mHealth)	Heart failure	Yes	Yes	N/A
23	Paré G Jaana M Sicotte C.;^290^	2007	16	Telemonitoring (mHealth)	Hypertension and heart disease	N/A	Yes	Yes
24	Shah A Clarke M Sharma U.;^291^	2011	13	Telemonitoring (mHealth)	Congestive heart failure	N/A	No	No
25	Rawstorn JC, Gant N, Direito A, Beckmann C, Maddison R.^151^	2016	11	Teleconsulting	Cardiac rehabilitation	Yes	N/A	N/A
26	Neubeck L Redfern J Fernandez R Briffa T Bauman A.^292^	2009	11	Telemonitoring (mHealth)	Coronary disease	Yes	N/A	N/A
**#ID**	**Early diagnosis**	**Increased adherence to therapy**		**Not conclusive**		**Cost reduction**	**Most frequent economic analysis method among articles**	
15	N/A	N/A		N/A		N/A	N/A	
16	N/A	N/A		N/A		Yes	Cost-effectiveness	
17	N/A	N/A		N/A		N/A	N/A	
18	Yes	NA		NA		N/A	N/A	
19	Yes	N/A		N/A		N/A	N/A	
20	N/A	N/A		N/A		N/A	N/A	
21	Yes	Yes		N/A		N/A	Cost-effectiveness	
22	Yes	N/A		N/A		No	N/A	
23	Yes	N/A		N/A		No	N/A	
24	NA	N/A		N/A		Yes	N/A	
25	Yes	N/A		N/A		N/A	N/A	
26	Yes	N/A		N/A		Yes	N/A	
27	Huang K Liu W He D Huang B Xiao D Peng Y.^301^	2015	9	Teleconsulting	Coronary disease	Yes	NA	NA
28	Purcell R McInnes S Halcomb EJ.^302^	2014	13	Telemonitoring (mHealth)	Chronic heart failure	Yes	Yes	NA
29	Chaudhry SI Phillips CO Stewart SS Riegel B Mattera JA Jerant AF Krumholz HM.^303^	2007	9	Telemonitoring (mHealth)	Heart failure	NA	Yes	Yes
30	Pfaeffli Dale L, Dobson R, Whittaker R, Maddison R.^304^	2016	7	Telemonitoring (mHealth)	Cardiovascular diseases	NA	NA	NA
31	Rush KL Hatt L Janke R Burton L Ferrier M Tetrault M.;^262^	2018	16	Tele-education	Chronic heart failure	NA	Yes	Yes
32	Hameed AS, Sauermann S, Schreier G.^305^	2014	9	Comparison between telephone teleconsultation services	Heart failure	NA	NA	NA
33	Feltner C Jones CD Feltner C Zheng ZJ Sueta CA Coker-Schwimmer EJ Arvanitis M.^259^	2014	47	Telemonitoring (mHealth)	Heart failure	NA	Yes	NA
34	Beatty AL, Fukuoka Y, Whooley MA.^150^	2013	3	Telemonitoring (mHealth)	Cardiac rehabilitation	NA	NA	NA
35	Driscoll A, Meagher S, Kennedy R, Hay M, Banerji J, Campbell D, Cox N, Gascard D, Hare D, Page K, Nadurata V, Sanders R, Patsamanis H.^258^	2016	29	Telemonitoring (mHealth)	Heart failure	Yes	Yes	Yes
**#ID**	**Early diagnosis**	**Increased adherence to therapy**		**Not conclusive**		**Cost reduction**	**Most frequent economic analysis method among the articles**	
27	Yes	N/A		N/A		N/A	N/A	
28	Yes	N/A		N/A		Yes	N/A	
29	N/A	N/A		N/A		Yes	Cost-effectiveness	
30	N/A	Yes		N/A		Yes	N/A	
31	Yes	Yes		N/A		Yes	N/A	
32	Yes	Yes		N/A		No	N/A	
33	N/A	N/A		N/A		N/A	N/A	
34	Yes	Yes		N/A		N/A	N/A	
35	N/A	N/A		N/A		N/A	N/A	

Among the studies, 26 evaluated the telemedicine telemonitoring service mHealth as primary intervention versus usual face-to-face treatment. According to the authors, the ease of use of mobile phones, through the use of applications for remote monitoring and quick communication with patients, was the most common.^271^

The most common outcomes in the evaluated studies were a reduction in mortality rate (54% of the reviews) and in the number of hospital readmissions (57% of the reviews), with at least one of these outcomes appearing in 26 studies. Other expected results were reduced hospitalization, early diagnosis, and better medication adherence.

On the other hand, economic evaluations were less frequent. Only 16 reviews found conclusive results with cost analyses, of which 13 concluded that telemedicine provided savings to the paying source by reducing costs. Eight reviews included the economic evaluation of incorporating telemedicine in cardiology, with cost effectiveness being the most frequent evaluation (seven studies), while cost minimization was included in a single study. One study suggested that the few existing economic assessments have low methodological rigor, not allowing an assertive conclusion about the economic viability of the implementation.^264,271^

Briefly, studies on heart failure telemonitoring have shown that support strategies (video conferencing or telephone) are cost effective, meaning, they have a potential for financial return. Studies evaluating monitoring by cardiac devices showed an incremental cost-effective ratio of US$ 13,979 per QALYs. In a meta-analysis, device telemonitoring was related to a 44% reduction in hospital visits, with no effect on mortality, but a 15-50% reduction in direct health costs.^272^ The economic results of noninvasive telemonitoring are even more heterogeneous. Some clinical trials have shown neutral results, while one showed a significant reduction in heart failure readmissions and a direct total cost reduction of € 3,546 per patient for 6 months of follow-up.^273^ In a Dutch clinical trial, the TEHAF trial, the likelihood of cost-effectiveness for remote monitoring was 48% (€ 50,000/QALY threshold), probably due to differences between institutions. One of the most detailed studies was developed for the UK health system perspective using a Markov model comparing usual treatment, telephone support, or remote telemonitoring for patients with heart failure after hospital discharge. Assuming monthly costs of £ 27 for standard care, £ 179 for telephone support, and £ 175 for telemonitoring during business hours, the most cost-effective strategy was telemonitoring, with values below £ 20,000 per QALY. The telephone support strategy was very unfavorable, with an ICER of £ 228,035/QALY compared with telemonitoring.^274^

Variables evaluating effectiveness repeat across the studies, notably the reduction in mortality and in hospitalization frequency. However, accurate cost collection methods, definition of which cost variables should be collected, and the application of economic models still lack standard recommendations in the literature. More than 70% of the studies did not consider at least one category: health care costs, patient and family expenses, or lost productivity. Many failed to include salaries and benefits, training time, amortized capital investments, data and follow-up operations, and overhead costs. Moreover, an important constraint in these economic analyses has been the great heterogeneity of technology (intervention) and even the control group (alternatives). Technologies supporting telemedicine services advance at an impressive pace and range from complex structures and large investments less than 10 years ago to cost-effective solutions based on cell phones and mobile devices.^256,263^ These are distinct services requiring a high initial investment, but most data point to a return on investment over time due to the volume of patients who then do not require the use of the traditional health care system.^260,269^ Economic assessment methods must, therefore, track the service over time, including the outcomes of patients receiving care or monitored by the telemedicine services. Given the diversity of the benefits gained, the applied cost methodology - dedicated data reflecting conditions of the local health care system - must be evaluated for a proper understanding of the cost effectiveness of these technologies.

### 5.4. Economic Evaluations of Telemedicine in Brazil

These new technologies are being gradually introduced in the routine of hospitals, clinics, and offices in the private and public sectors in Brazil. The first of these technologies to be applied was the transmission of ECG data for remotely reporting. In 1994, the company Telecardio started using this technology, transmitting the tests by telephone, and in 1995, the *Instituto do Coração* (InCor) created a service called ECG-FAX. Later, in 2005, a telecardiology system or Minas Telecardio project was implemented at UFMG’s Clinics Hospital.^275^ In 2007, the Ministry of Health, aiming to develop actions to support primary care teams through permanent education and virtual technologies, created the *Telessaúde Brasil* program, later renamed *Telessaúde Brasil Redes*. Nine Brazilian Telehealth Centers (*Núcleos de Telessaúde no Brasil*) were initially established, offering teleconsulting (consultation between professionals) and telediagnosis (ECG) in the public sector. Currently, several companies offer remote reporting of ambulatory BP monitoring (ABPM), Holter, and remote echocardiography and imaging analysis. AI is currently used in teleconsulting and is at an advanced stage in the preparation of diagnostic test reports. Telemonitoring of patients with heart failure is also ongoing in Brazil.

The routine use of these technologies in Brazil is an indirect indication of their effectiveness, and the continuing operation of companies in this sector is an indirect measure of cost-effectiveness, although there are few formal studies on this subject in Brazil.

At least two initiatives for the application of new technologies in cardiology in Brazil focused on these cost-effectiveness studies: 1) the telemedicine service and remote patient monitoring of InCor at the University of São Paulo Medical School and 2) the telediagnosis and teleconsultation system of the Minas Gerais Teleasssistance Network (RTMG) of the Clinics Hospital at UFMG.

In addition to the results from telecardiology services, an analysis of savings estimates for the state of Rio Grande do Sul through the *TelessaúdeRS* project, which offers 20 clinical specialty teleconsultation services, was conducted and are presented in this chapter as a third approach to an economic evaluation of telemedicine in Brazil.

### 5.5. InCor-FMUSP Telemedicine and Patient Monitoring Service

Stevens et al. evaluated the economic burden and impact on patients’ disability of four main heart conditions - heart failure, AMI, AFib, and hypertension - in 2015 in Brazil.^276^ Specifically for hypertension, the authors assessed the cost effectiveness of conventional care versus telemedicine and structured telephone support over a 30-year time horizon after 2015. A summary of the results found in the study is shown in Table 5.2.

**Table 5.2:** Results of comparison: treatment of hypertensive patients with conventional care through Telemedicine and through a structured telephone support system over a 30-year timeframe from 2015

	Conventional care	Telemedicine	Structured telephone support
Total cost (R$)	5,832	55,930	49,870
Incremental cost (R$)		50,098	44,038
Additional life-years^1^		1.89	1.61
Cost per additional life-year (R$/year)		26,437	27,281

1 Additional life-years reflect the impact of longevity on the patient's quality of life.

Both technologies were considered cost effective by the authors, assuming the standard defined by the WHO of an intervention being considered cost effective when having a cost per life-year between one and three times the gross domestic product per capita per QALY. However, the Brazilian health authorities have not yet defined the country’s ICER. Thus, safe inferences cannot be made on whether the procedures applied in telemedicine within the SUS would be cost effective or not within these scenarios. According to Brazilian law, products, medications, or procedures included in SUS’ protocols must be evaluated for safety, efficacy, effectiveness, and cost effectiveness. Therefore, an effective reference is still needed to validate the economic assessment in question.^277^

### 5.6. Telediagnosis and Teleconsultation System of the Minas Gerais Teleassistance Network (Rede de Teleassistência de Minas Gerais, RTMG) at the Clinics Hospital of UFMG

The Telehealth Center (*Centro de Telessaúde*, CTS HC/UFMG) coordinates the RTMG, a collaborative network established in 2005 by seven public universities in Minas Gerais: UFMG, Federal University of Uberlândia (UFU), Federal University of Triângulo Mineiro (UFTM), Federal University of Juiz de Fora (UFJF), Federal University of São João Del Rei (UFSJ), State University of Montes Claros (Unimontes), and Federal University of Jequitinhonha and Mucuri Valleys (UFVJM).

Telecare activities include teleconsultation and telediagnosis. Teleconsultations are mostly asynchronous through regulatory calls in medicine, nursing, dentistry, physiotherapy, pharmacy, psychology, nutrition, and speech therapy. Telediagnosis consists of the analysis and reports of ECGs, ABPM, Holter, and retinography, along with synchronous cardiology teleconsultations to support critical clinical cases. The service is registered in the CRM of the State of Minas Gerais.

The RTMG activity was initiated with the research project Minas Telecardio in 2006, implementing a telecardiology service with ECG reports and teleconsultation in 82 municipalities in Minas Gerais. The RTMG activities expanded over time and currently connects 814 municipalities with more than 1,000 telehealth units in Minas Gerais. Within the ONTD project of the Ministry of Health, it began to offer nationwide telecardiology services on September 2017, and currently serves 90 municipalities in the states of Acre, Bahia, Ceará, Mato Grosso and Roraima. This expansion was partly the result of studies proving the cost effectiveness of the system for the main RTMG funders (Ministry of Health and Minas Gerais State Department of Health).

Using the results of the Minas Telecardio Project, Andrade et al.^278^ compared the cost-benefit ratio of remote ECG reporting, considering the hypothesis of economic benefit in performing ECGs in the telecardiology project compared with the referral of the patient to perform ECG examination at another location.^278^ The study was conducted between June 2006 and November 2008 in 82 municipalities in rural areas of the state of Minas Gerais. Each municipality received a microcomputer with a digital electrocardiograph machine and had the possibility of forwarding the ECG recordings and establishing communication with the cardiology department at university hospitals of the RTMG. The costs of the project were divided into two categories: related to the implementation and related to the maintenance of the telecardiology system. The cost of moving patients was assessed, including the cost of transportation (using city-provided resources), the cost of food during their absence from home, and the cost of a missed working day (both paid by the patient). The cost-benefit without inclusion (perspective of the public health service) and with inclusion (perspective of the society) of the patients’ costs were evaluated. For the face-to-face scenario, the cost of ECG and cost of the consultation were added (R$ 5.15 and R$ 10.00, respectively, based on the SUS’ reference table). The sources of data for the analysis were mainly the National Household Sample Survey (*Pesquisa Nacional de Amostra de Domicílios*, PNAD), SIA-SUS Ambulatory Information System, and CNES.

Considering the cost of implementation and maintenance of the project of R$ 1,818,282.87 and the number of examinations performed in the 30-month period (62,865 examinations from August 2006 to December 2008), the unit cost of each remote report was R$ 28.92. A summary of the results is shown in Table 5.3.

**Table 5.3:** Comparison of costs between the alternatives remote report and face-to-face report in the Minas Telecardio Project^278^

Scenario	Cost (R$)	Difference (R$)
1. Remote report	28.92	
2. Face-to-face report without costs of patient displacement	30.91	1.99
3. Face-to-face report with costs of patient displacement	49.83	20.91

A sensitivity analysis showed that the results are sensitive to patient travel costs, particularly related to the driver’s salary and number of patients per vehicle. Given the small difference between scenarios 1 and 2, it can be concluded that, in some situations, telecardiology may not be more economical from the point of view of public health service.

At that time, the system had a relatively low output and, since the activities have a high fixed cost, resulted in a high cost for the remote report. With the expansion of the system to other municipalities, the cost of the activities reduced, increasing the cost effectiveness of the system.

In 2007, the Ministry of Health, with resources from PAHO, engaged the CTS HC/UFMG in the project “Analysis of the Financial Management of Telehealth Services Applied to Primary Care.” In the project, the analysis of the economic sustainability of the application of telehealth in primary care was based on a comparison of costs between two scenarios:

- Face-to-face care: when the patient is treated in primary care and requires to be subsequently referred to secondary level care;
- Remote care: when the primary care physician receives remote support through a telehealth service, and this support avoids the referral of the patient.

The results refer to 20 municipalities participating in the National Telehealth Project with reliable data, located in the North/Northeast regions and Jequitinhonha Valley in Minas Gerais, considered one of the poorest regions of the state. The main results of the project are shown in table 5.4.^279,280^

**Table 5.4:** Comparison of average costs (R$/month/municipality) between face-to-face and remote care in the project "Analysis of Financial Management of Telehealth Services Applied in Primary Care"^279,280^

Cost item (R$/month)	Face-to-face care	Remote care
Patients referral	2,399.58	697.78
System implementation		56.03
Equipment depreciation in the municipality		101.97
Equipment maintenance in the municipality		40.79
Capital cost of equipment		51.92
Activities of remote care*		210.80
Total	2,398.58	1,159.29

* ECG report, case discussion and teleconsultation.

This project presented more realistic results compared with the previous one, as it collected information about the cost of patient referral directly in the municipalities. However, it maintained the sample of the participating municipalities concentrated in the same region of the state, the Jequitinhonha Valley, which has a low human development index (HDI). In 2009, the Minas Telecardio Expansion project expanded the service to higher HDI regions for a final sample of 66 other municipalities. The results were similar to those of the previous project and are shown in table 5.5.^281-283^

**Table 5.5:** "Economic Analysis and Impact of the Application of Telehealth Services in Primary Care in Municipalities of Minas Gerais"^282^

Average cost of each telehealth activity in 2010	R$ 10.68
Average fixed cost of patient referral	R$ 41.77
Savings to the municipality by avoided referral	R$ 71.11
Minimum monthly number of referrals reduction per municipality to enable the system	4.28
Minimum monthly number of activities per municipality to enable the system	5.5
Average monthly number of activities by municipality in 2010	28.5
Investment by SES/MG (2005-2009)	R$ 11,599,638.00
Savings for the health care system (from June 2006 to July 2011)	R$ 31,970,549.13
Return on Investment (ROI) (savings:investment)	2.76:1
% of referrals avoided	78%
Minimum referral distance for system viability (approximate)	54 Km
Average cost of each telehealth activity in 2010*	R$ 10.68

* ECG report, case discussion and teleconsultation.

Recently, a study (pending publication) was conducted to assess the cost effectiveness of the ONTD. The ONTD is a project of the Ministry of Health to offer telecardiology services (ECG reports and teleconsultations) to all Brazilian states, for which the CTS HC/UFMG was chosen as Specialist Center, that is, the service provider. State Telehealth Centers receive funding from the Ministry of Health to train and implement care in municipalities of their state, previously agreed with the State Health Departments, which may forward 24/7 their test requests and teleconsultations to the Expert Center. The requests may be elective or urgent. The report is available on a platform, and, when necessary, local physicians can ask questions as part of teleconsultation. An alert system informs physicians and nurses about critically emergent situations. The effectiveness of the system has been proven by performance indicators (time to submit tests, waiting time for analysis of urgent/elective reports, time to the first analysis of the report, number of tests requested by the municipality, etc.), and user satisfaction. The results proved the effectiveness of the system by replacing alternatives previously available to obtain the test/report (referral of the patient, periodic visits of the cardiologist to the municipality, and outsourcing of tests to private companies/clinics). In terms of these alternatives, the cost of the test by the ONTD is about five times lower, demonstrating cost effectiveness.

### 5.7. Analysis of the Economic Impact of the TelessaúdeRS Teleconsulting Service

An economic analysis of the TelessaúdeRS service was performed to evaluate the financial results of the service generated to the state. Teleconsultations are offered for various specialties, in addition to the contribution to the regulation of waiting lists for the state of Rio Grande do Sul. Teleconsultations in endocrinology, gastroenterology, proctology, rheumatology, and urology were selected as samples.

The cost per teleconsultation (R$ 110.29) was evaluated considering data from 8 months of service and included costs of the physical structure of the service (rent, energy, depreciation, server capacity, among others) and payment of professionals. As a premise, all teleconsultations and regulations that resulted in the cancellation of the face-to-face consultation would represent, for the state, savings in patient transportation to Porto Alegre (which is variable according to the municipality of origin) and payment of face-to-face consultation to the municipality (R$ 100.00). Estimated savings were calculated as the difference between the cost of performing canceled teleconsultations and the cost of face-to-face care.

The analysis identified that over 21 months (October 2016 to June 2018), the state saved R$ 2,287,121.78, of which 47% were related to consultations and 53% to transportation services. For teleconsultation, the service was found to be attractive up to the amount of payment per consultation of R$ 38.95, considering the average cost of transportation. The municipalities with the greatest distances from Porto Alegre had higher savings, except for four municipalities neighboring Porto Alegre, which had a higher number of avoided teleconsultations than the others’ average.

Of note, a comparison of these results with those of other states and services should consider that the services of Telessaúde are provided by scholars and “CLT” professionals (employees following the Brazilian Labor Laws); therefore, Telessaúde can operate at a lower cost per teleconsultation. The operation of a similar service including only “CLT” employees would increase the cost per each teleconsultation and require additional economic analysis.

The WHO considers cost effective those interventions with ICER between 1 and 3 times the gross domestic product per capita per life-year, unless adjusted for QALY.

## 6. Recommendations

The Brazilian Society of Cardiology, due to the growing interest in the use of telemedicine for the expansion of health care, particularly in the area of cardiology, prepared this guideline to inform the medical category and society in general on the scientific and technological basis of telemedicine applications considering the current scenario.

Even though there is growing enthusiasm for the democratization of information and communication technologies, it is important to point out that barriers to implementation persist across the country and must be addressed. The most significant ones are:

- update of laws and regulations applicable by the health authorities and CFM;
- provision of minimum telecommunications infrastructure in health care facilities, especially in remote areas;
- cost of technology;
- need for qualification and training of human resources;
- incorporation of technologies in the SUS’ public policy list and in the List of Procedures and Events in Health of the National Health Agency.

By bringing to light the discussion about telemedicine applications, in addition to its media repercussion, we seek to provide scientific and technical support for the elaboration of health care policies consistent with the use of this technology. In this sense, we must formally incorporate, after due evaluation by the CONITEC, the various possibilities available today linked to the respective clinical protocols and therapeutic guidelines (PCDT). Also, in the context of supplementary health, it is necessary to include in the List of Procedures and Events in Health of the National Health Agency those with scientific recognition and authorized for current use in the country.

As discussed in this guideline, with rare exceptions, there is no provision in the Brazilian Hierarchical Classification of Medical Procedures (which is a condition for inclusion in the NHA coverage list) for common procedures in telemedicine. Generic coding is used, with descriptions that are broad in nature and allow for likelihood use, such as code 2.01.01.20-1 (clinical and electronic evaluation of a patient with a cardiac pacemaker or resynchronization defibrillator or defibrillator). However, the reimbursement of this service will depend on the health care provider’s sole decision. This fact limits the applicability of telemedicine in the field of supplementary health, with the related consequences.

In 2015, the Brazilian Society of Cardiology, through the Telecardiology Guideline for the Care of Patients with Acute Coronary Syndrome and other Heart Diseases,^133^ made recommendations on this topic. However, the current version of the broader guideline addresses new applications for telecardiology, especially those already incorporated into the health care system. It still deals with future perspectives, such as the use of telerobotics and AI. The authors, focusing on current scientific evidence and cost effectiveness, have updated the recommendations to guide public and private health care providers on judicious use of telemedicine applications in Brazil.

Table 6.1 summarizes the recommendations outlined in this guideline.

**Table 6.1:** Recommendations for the practice of Telemedicine in Brazil

Clinical indication	Class of indication	Level of evidence	references
**Teleconsultation**			
Teleconsultation assists general practitioners from remote areas in the clinical evaluation of patients with suspected or established cardiovascular disease, being cost-effective from the SUS perspective	IIa	B	^76,99,100,101,278,279^^,^^280,281,282^
Teleconsultation assists physicians working in emergency care in the management of cases of acute cardiovascular diseases	IIa	C	^133,134,137,138,139,140^^,^^141,142,143,144,145,149^
Teleconsultation assists in regulating access to specialized care in patients with suspected or established cardiovascular disease	IIa	C	^101,102,103,104^
**Telediagnosis**			
Tele-electrocardiography is a feasible and effective alternative to offer electrocardiography in health care systems, and is particularly useful and cost effective in primary care and remote locations	I	B	^61,105,106,107,278^
Telemedicine with transmission of electrocardiographic report in pre-hospital care of patients with suspected acute myocardial infarction reduces cardiovascular outcomes and early and late mortality	I	B	^87,137,138,139,140,141^^,^^142,143,144,145^^,^
Tele-echocardiography with teleconsultation is effective in the early detection of congenital heart disease in newborns	IIa	B	^182,183,306^
Tele-echocardiography allows the early detection of subclinical cases of rheumatic heart disease in children and adolescents	IIa	B	^111,112,307^
Tele-echocardiography in primary care allows early detection of cases of heart disease and may help prioritize referrals to specialized care.	IIb	C	^109,110,112,114^
Transmission of tomography and cardiac resonance imaging by telemedicine can be performed:			
- to obtain a second opinion	IIa	C	^190,192^
- for discussion in "Heart Teams"	IIa	C	^190,192^
- for remote support in emergency cases	IIa	C	^190,192^
- in sporadic routine cases	IIa	C	^190,192^
- for routine transmission of all cardiovascular imaging tests to specialized centers or groups	IIb	C	^190,192^
**Telemonitoring**			
Self-monitoring of blood pressure with telemonitoring helps in treatment control and adherence effective in reducing hospitalizations due to heart failure	IIa	B	^75,77,78,127,128,129,130^
Noninvasive telemonitoring strategies with structured telephone support are effective in reducing hospitalizations due to heart failure	I	A	^115,116,118,119,120,123^^,^^124,125,126,158,159,161^
Noninvasive telemonitoring strategies with structured telephone support are effective in reducing mortality in heart failure	IIa	A	^115,116,118,119,120,123^^,^^124,125,126,158,159,161^
Remote monitoring of patients with arrhythmias and implantable electrical devices, in addition to regular telemetric assessments, is effective in reducing outpatient visits and early detection of device dysfunction.	IIa	B	^157,160,162,163,164,165^^,^^166,167,168,169,170,174^^,^^175,177,178,179,180^
Telerehabilitation of eligible patients with heart failure, with or without left ventricular dysfunction, with NYHA functional class I-III, is effective in improving program adherence, quality of life and performance in the 6-minute walking distance test	IIa	B	^148,149,150^^151,152^
Private communication to send data or ask questions between physicians, in closed groups of specialists, or among the clinical staff of an institution or chair, safeguarding professional confidentiality	I	C	^191^
Private communication between physicians and patients through communication platforms to send data or ask questions, safeguarding professional confidentiality	IIa	C	^191^
